# Enhancing biometric identification using 12-lead ECG signals and graph convolutional networks

**DOI:** 10.3389/fdgth.2025.1547208

**Published:** 2025-04-08

**Authors:** Maram Al Alfi, Pedro Peris-Lopez, Carmen Camara

**Affiliations:** Computer Science and Engineering Department, University Carlos III of Madrid, Madrid, Spain,

**Keywords:** graph convolutional networks (GCN), electrocardiogram (ECG), mutual information (MI), Identification, 12 ECG leads

## Abstract

**Introduction:**

The electrocardiogram (ECG) is a highly secure biometric modality due to its intrinsic physiological characteristics, making it resilient to forgery and external attacks. This study presents a novel real-time biometric authentication system integrating Graph Convolutional Networks (GCN) with Mutual Information (MI) indices extracted from 12-lead ECG signals.

**Methods:**

The MI index quantifies the statistical dependencies among ECG leads and is computed using entropy-based estimations. This index is used to construct a graph representation, where nodes correspond to ECG features and edges reflect their relationships based on MI values. The GCN model is trained on this graph, enabling it to learn complex patterns for user identification efficiently.

**Results:**

Experimental results demonstrate that the proposed GCN-MI model achieves 100% accuracy with a 5-layer architecture at a k-fold of 75, outperforming conventional approaches that require less training data.

**Discussion:**

This work introduces several innovations: the integration of MI indices enhances feature selection, improving model robustness and efficiency; the graph-based learning framework effectively captures both spatial and statistical relationships within ECG data, leading to higher classification accuracy; the proposed approach offers a scalable and real-time biometric authentication system suitable for applications in finance, healthcare, and personal device access. These findings highlight the practical value of the GCN-MI approach, setting a new benchmark in ECG-based biometric identification.

## 1 Introduction

The global cost of data breaches has reached staggering figures in recent years, with annual financial losses amounting to billions. These breaches expose sensitive information, including financial records, personal credentials, and medical histories, making traditional authentication methods such as passwords, PINs, and security tokens increasingly vulnerable. Conventional security mechanisms struggle to provide adequate protection as cyber threats become more sophisticated, leveraging phishing, social engineering, and AI-driven attacks. This growing crisis has accelerated the demand for biometric authentication systems, which leverage unique biological traits that are inherently more secure and resistant to traditional attack vectors. However, existing biometric technologies, such as facial recognition and fingerprint scanning, are not immune to vulnerabilities. Spoofing attacks, sensor noise, environmental dependencies, and potential privacy concerns limit the reliability of these modalities, necessitating the development of alternative biometric modalities that offer higher resilience, accuracy, and security. Electrocardiogram (ECG)-based authentication emerges as a promising solution due to its physiological uniqueness, internal nature, and resistance to forgery. Nevertheless, designing effective ECG biometric systems remains challenging due to data scarcity, inter-user variability, and computational efficiency constraints. This study introduces a novel approach that integrates Graph Convolutional Networks (GCN) with Mutual Information (MI) indices to overcome these challenges and significantly enhance the robustness of ECG-based biometric authentication.

The most appropriate and adaptable biometric characteristics include facial features, fingerprints, palm prints, voice, electrocardiograms (ECG), and iris patterns (1). Each of these modalities has its strengths and weaknesses. For instance, facial recognition and fingerprint systems are widely adopted due to their ease of implementation and the availability of standard imaging devices. However, these methods are not without limitations. They are susceptible to spoofing attacks, where counterfeit replicas such as fake fingerprints or photos of faces are used to bypass security measures. Additionally, environmental factors, user behavior, or physical changes such as aging, scarring, or injuries can degrade the accuracy and reliability of these systems over time (2).

In contrast, ECG presents a compelling alternative as a biometric modality. Unlike external traits such as fingerprints or facial features, which can be replicated or influenced by external conditions, ECG signals are internal physiological characteristics inherently linked to an individual's unique cardiac activity. This makes ECGs significantly more difficult to forge or manipulate. Furthermore, ECG-based systems are resilient to many environmental and physical factors affecting other biometric modalities. For example, while a fingerprint scanner might fail if the user's hands are dirty or injured, an ECG system can continue to function reliably as it measures electrical activity from within the body. These advantages position ECG as a highly secure and robust biometric characteristic, particularly in contexts where high levels of security and reliability are essential. By addressing the limitations of traditional biometric systems and meeting the growing demands for enhanced authentication methods, ECG-based technologies are playing a pivotal role in the evolution of biometric security systems.

A significant amount of research is currently being conducted on machine learning and deep learning approaches for ECG-based biometric detection (3). These advanced algorithms demonstrate significant promise in improving the accuracy of biometric authentication systems. Nevertheless, numerous studies have encountered difficulties due to the limited data available—primarily ECG beats gathered from the same participants—which often restricts the models' generalization and performance (4).

Convolutional neural networks (CNNs) are a common deep learning technique. They are vital tools that significantly boost diagnosis accuracy in many medical and biometric applications (5). Feeding these networks with ECG signals that contain unique information about the heart's electrical activity and exhibit significant characteristics across multiple angles or leads enhances the accuracy of arrhythmia detection (6).

**Figure 1:**
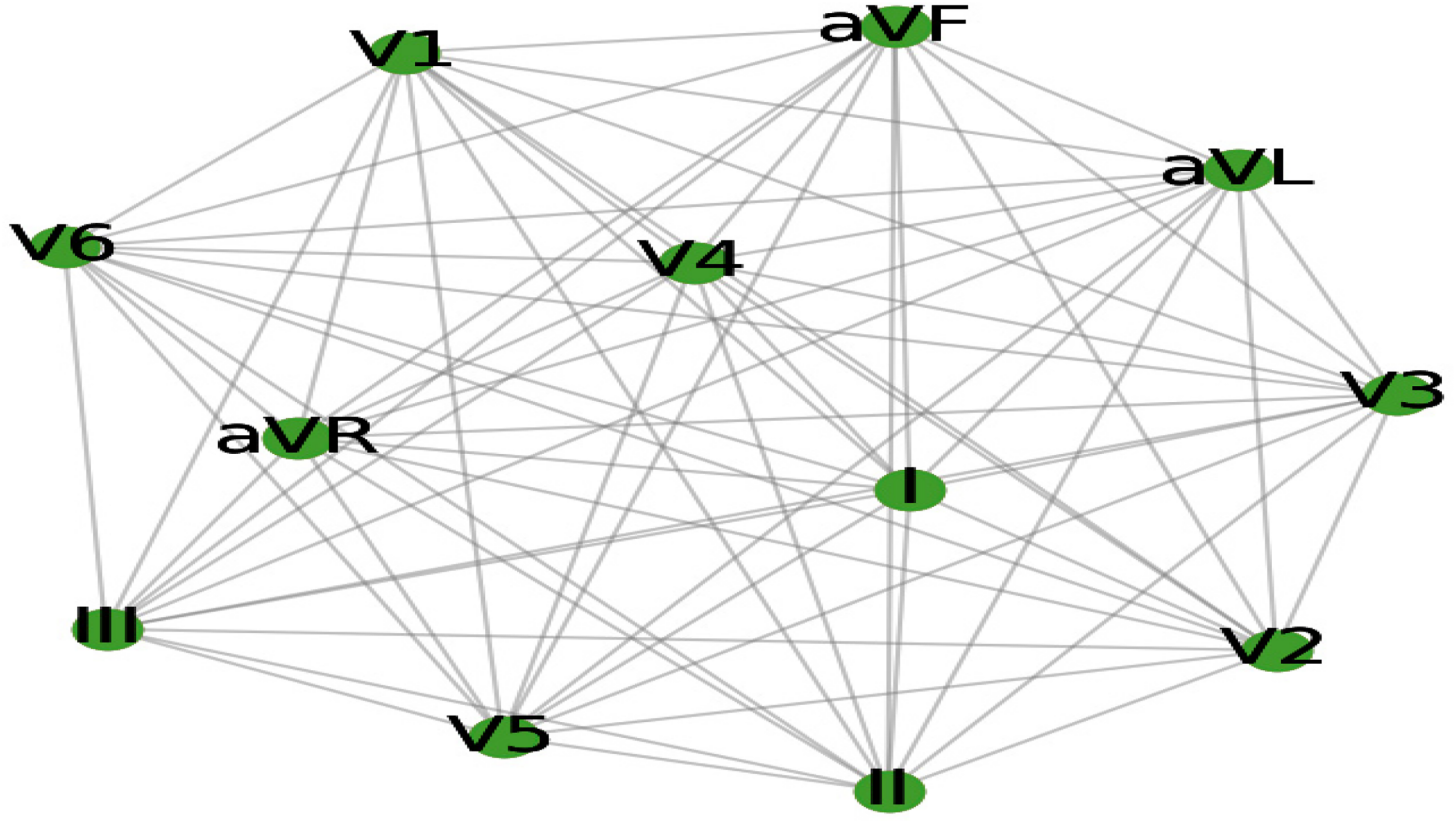
The 12-lead ECG electrical activity is represented as a graph.

According to Zhang et al. (7), graph convolutional networks (GCNs) are a cutting-edge deep learning technique explicitly designed to process and analyze graph-structured data, in contrast to conventional convolutional neural networks (CNNs), which are typically limited to processing grid-like structures, such as pixel images. The complex relationships and interactions between entities, represented as nodes, can be captured and utilized by Graph Convolutional Neural Networks (GCNs). Using a graph composed of these nodes and edges, complex real-world events, such as social networks and molecular structures, as well as the heart's electrical activity as depicted in a 12-lead ECG (see Figure 1), can be represented (6).

One of the key advantages of GCNs over CNNs is their flexibility in handling irregular and non-Euclidean data, where the relationships between data points are not arranged in a fixed grid. CNNs typically rely on fixed square kernels to perform convolutions, which are well-suited for image data but less effective for more complex, irregular structures, such as graphs (8). On the other hand, GCNs can apply convolutions to any network structure, capturing interactions between closely located nodes, even in non-uniform or randomly distributed locations. This makes GCNs particularly beneficial for applications that display data as graphs, enabling them to learn the underlying structure more effectively.

In the context of this study, ECG data are modeled as a graph, where each node represents one of the heart's 12 leads, and the edges capture the relationships or dependencies between these leads. The electrical potential recorded by each lead is defined as a time series, and knowing the relationships between these time series is crucial for successfully authenticating individuals based on their ECG patterns. Autocorrelation indices measure the strength of correlation between a time series and its lagged copy, thereby assessing these dependencies. Additionally, Pearson correlation indices can be employed to evaluate the linear relationships between the ECG signals recorded by different leads, providing a more detailed understanding of the underlying structures in the data (9). However, ECG signals often exhibit both linear and nonlinear dependencies, which Pearson correlation alone cannot fully capture. To address this, mutual information (MI) is introduced as a complementary measure, which utilizes entropy to quantify the amount of shared information between two time series. Unlike Pearson correlation, MI can detect linear and nonlinear relationships, making it a powerful tool for analyzing the complex dependence structures inherent in ECG data (10). By merging MI, the GCN can more accurately capture the rich, multifaceted relationships between the ECG leads, enhancing its ability to learn from the data and improve the performance of the biometric identification system.

This study's combination of GCNs and mutual information creates a robust framework for modeling the interrelationships between ECG leads. By treating the ECG signals as a graph and using MI to define the edges between nodes, the GCN-based model can leverage the shared information between the leads to more accurately identify individuals based on their unique ECG patterns. This approach improves the accuracy of the biometric identification system. It provides a more comprehensive understanding of the complex dependencies within the ECG data, offering a significant advancement in the field of ECG-based biometric authentication. A graph was constructed to efficiently train the GCN to represent the interrelationships between the ECG leads using the mutual information (MI) index, which quantifies linear and nonlinear correlations. This graph-based representation captures the intricate dependencies across the 12 leads, allowing the GCN to learn from the shared and complementary information inherent in the ECG signals.

By leveraging the GCN-MI model developed in this study, individuals can be accurately identified and verified using their unique biometric signatures derived from 12-lead ECG data. The MI index is crucial in capturing the subtle patterns and relationships between the leads, often missed by simpler models that treat each lead independently. This enables a more comprehensive and robust approach to biometric authentication, allowing the system to differentiate between individuals with greater precision.

Table 1 compares various methods for ECG-based user authentication using different datasets and similarity measures. The results in Table 1 validate MI as the most effective parameter for feature extraction and graph construction, outperforming traditional measures such as Pearson correlation, Euclidean distance, and cosine similarity, with 100% accuracy on the INCART database. Its ability to capture complex relationships in ECG signals justifies its selection in our proposed method.

**Table 1:** Performance comparison of the proposed method with other approaches.

Method	Dataset	Parameter used	# ECG Leads	Accuracy (%)	Ease of Spoofing
Barros et al. (11)	PhysioNet	Euclidean distance	1	80.00	High
Zhang et al. (12)	PhysioNet	Euclidean distance	2	97.60	High
Ibtehaz et al. (13)	ECGID	Cosine similarity	3	98.71	High
Prakash et al. (14)	ECGID	Euclidean distance	2	99.85	High
Agrawal et al. (15)	PTBDB	Pearson correlation	1	98.30	High
Wang et al. (16)	ECGID	Pearson correlation	2	98.25	High
**Proposed method**	**INCART**	**MI**	**12**	**100** **.** **00**	**Low**

We can justify the use of MI in our proposed method by the following:

1. **Captures Nonlinear Dependencies:** Unlike Pearson correlation (used by Agrawal et al. and Wang et al.), which measures linear relationships, MI captures both linear and nonlinear dependencies, making it more effective for complex ECG patterns.
2. **Better Discriminative Power**: Compared to Euclidean distance (Barros et al.), which considers only spatial differences, MI measures the amount of shared information between signals, making it robust to amplitude variations and noise.
3. **Improved Similarity Measurement:** Cosine similarity (Ibtehaz et al.) focuses on directional similarity but may not capture all statistical dependencies in ECG signals. MI considers the probabilistic relationship, resulting in improved classification performance.
4. **Higher Accuracy on INCART DB:** The 100% accuracy achieved by using MI suggests its effectiveness in constructing the adjacency matrix for deep learning-based authentication, proving its superiority over previously used metrics.

The primary contribution of this study lies in introducing the GCN-MI approach as an innovative method for user identification and authentication, specifically designed to capitalize on the dense and interconnected structure of 12-lead ECG data. Conventional biometric methods, such as fingerprint or facial recognition, rely on external physical traits that can be susceptible to spoofing and environmental disruptions (17). In contrast, ECG signals offer a more profound, physiological-based biometric feature that is more resistant to forgery or external manipulation (18). By integrating MI-based graph convolution into the ECG-based biometric system, this study enhances the system's ability to capture and utilize complex data relationships, resulting in a more nuanced, reliable, and accurate identification process. The implications of this approach would be far-reaching.

By demonstrating that incorporating complex inter-lead relationships via MI can significantly improve the accuracy of biometric systems, the study paves the way for deploying more secure and reliable applications in critical sectors. For instance, in the financial industry, where secure user identification is crucial, ECG-based biometric authentication can provide an additional layer of protection against identity theft and fraud. Similarly, ECG-based identification can be used in healthcare to securely verify patients, ensuring that medical records and treatment plans are accurately matched to the correct individual. In personal security, ECG-based biometrics could offer a more secure alternative to accessing sensitive systems or information.

Ultimately, this study demonstrates the advantages of incorporating complex data relationships into biometric authentication systems. By focusing on the intricate connections within 12-lead ECG data, the GCN-MI approach represents a significant advancement in biometric identification, offering a more accurate and secure solution. This creates new opportunities for developing sophisticated biometric systems that can be applied in real-world situations, thereby enhancing security and reliability in various sectors and applications.

The primary contributions of this work are summarized as follows:

1. Innovation in the design approach: This study introduces a novel GCN-MI framework, which models the statistical dependencies between ECG leads using Mutual Information (MI) indices. This significantly enhances biometric feature selection and reduces feature redundancy.
2. Theoretical advancements: Information-theoretic justifications support the integration of MI indices into the GCN architecture, demonstrating their effectiveness in capturing both linear and nonlinear dependencies within ECG signals and improving user differentiation.
3. Optimization and computational efficiency: The proposed system utilizes an efficient adjacency matrix construction based on mutual information (MI) values, thereby significantly reducing computational complexity compared to traditional deep learning-based ECG authentication methods.
4. Enhancements in model architecture and performance: The GCN-MI model outperforms conventional ECG biometric techniques, achieving 100% accuracy with a 5-layer architecture at k-fold 75 while requiring less training data. The framework offers a scalable and real-time biometric authentication solution suitable for finance, healthcare, and secure access system applications.

These contributions establish a new benchmark for ECG-based biometric identification, demonstrating the practical applicability of GCN-MI in high-security authentication scenarios.

## 2 Related work

Recent research on ECG-based authentication has made significant advancements, leveraging deep learning techniques to address traditional challenges such as poor generalization, noise susceptibility, and dataset limitations. These studies primarily explore two major categories: (A) conventional machine learning techniques [Hammad et al., 2019; (2, 19)] and (B) deep learning-based methods (20, 21).

The application of machine learning for human authentication, mainly using ECG signals, is gaining momentum [Hammad et al., 2019; (22, 23)]. However, this approach has revealed several shortcomings that limit its effectiveness: poor generalization to unseen data, susceptibility to noise and artifacts in ECG signals, and the need for large datasets to achieve reliable performance. For example, manual feature extraction is sometimes necessary in traditional machine learning algorithms, which may not fully capture the complexity of ECG data, leading to reduced accuracy in real-world scenarios (24). Similarly, deep learning models, though more robust in feature learning, are computationally expensive and can suffer from overfitting, especially when trained on small datasets (25). Furthermore, variability in ECG signals due to physiological conditions, electrode placement, and emotional state presents an ongoing challenge, necessitating more sophisticated models or hybrid approaches that combine the strengths of multiple techniques (26).

In the following sections, we provide a detailed description of the latest research in this field.

### 2.1 Traditional machine learning approaches

Traditional machine learning techniques such as Support Vector Machines (SVM), k-nearest Neighbors (k-NN), and Random Forests have been widely used in ECG-based authentication [Hammad, M., Luo & Wang, 2019; (2, 19)]. These models typically rely on handcrafted feature extraction techniques, such as heart rate variability analysis, wavelet transforms, and statistical feature selection. While these methods can achieve reasonable accuracy, they are often constrained by their reliance on manual feature engineering, susceptibility to noise, and limited scalability to large datasets. Furthermore, they struggle to capture the complex spatial and temporal relationships within ECG signals (24), resulting in performance degradation when applied across diverse subjects and datasets.

### 2.2 Deep learning-based methods

Deep learning has significantly advanced ECG-based authentication by enabling automatic feature extraction and high-performance classification. The primary architectures used in this domain include Convolutional Neural Networks (CNNs), Recurrent Neural Networks (RNNs), Graph Convolutional Networks (GCNs), Transformers (27), and Generative Adversarial Networks (GANs) (28). Each approach offers distinct advantages and limitations:

#### 2.2.1 Convolutional neural networks (CNNs) for feature extraction

CNNs have been widely adopted in ECG classification and biometric authentication because they can extract spatial features from raw ECG signals without requiring manual feature engineering (29). These models capture local dependencies and morphological variations in ECG waveforms by leveraging convolutional layers. However, CNNs are limited in modeling long-term temporal dependencies, as they primarily focus on spatial feature extraction, making them less effective for tasks requiring sequential analysis (2).

#### 2.2.2 Recurrent neural networks (RNNs) for temporal dependencies

RNNs, particularly Long-Short-Term Memory (LSTM) and Gated Recurrent Unit (GRU) networks have demonstrated strong performance in ECG authentication by effectively capturing short—and long-term dependencies in time-series data. Unlike CNNs, which primarily focus on local feature extraction, RNN-based models learn sequential dependencies in ECG waveforms, making them suitable for applications where variations in heartbeat over time play a crucial role in classification (30, 31). However, RNNs have certain drawbacks, including high computational costs and difficulty training over long sequences, which can limit scalability and real-time applicability.

#### 2.2.3 Graph convolutional networks (GCNs) for graph-structured ECG data

GCNs have emerged as a promising deep learning technique for ECG-based biometric authentication due to their ability to process non-Euclidean structured data, such as the complex interdependencies among multiple ECG leads (32, 33). Unlike CNNs and RNNs, which assume a grid-like or sequential data structure, GCNs model the statistical dependencies between ECG leads as graph structures, allowing the network to capture spatial and statistical relationships within ECG signals. Studies such as our proposed GCN-MI method demonstrate that GCNs enhance classification accuracy and generalization capabilities in ECG biometric authentication by leveraging mutual information (MI) indices to define graph edges.

#### 2.2.4 Emerging technologies: transformers and GANs in ECG processing

Recent advances in deep learning have led to the exploration of Transformers and Generative Adversarial Networks (GANs) in ECG-based authentication.

A. **Transformers:** Unlike CNNs and RNNs, Transformers utilize self-attention mechanisms to capture long-range dependencies in ECG signals, thereby enhancing model interpretability and robustness (34, 35). Their parallel processing of entire ECG sequences makes them more computationally efficient than traditional sequential models.
B. **GANs**: Generative Adversarial Networks have been applied to generate synthetic ECG signals, improving model training and generalization when working with limited datasets. These models have been instrumental in mitigating data scarcity issues by augmenting ECG datasets with realistic synthetic samples (36–38), enhancing the robustness of authentication models.

## 3 Proposed methodology

**Figure 2:**
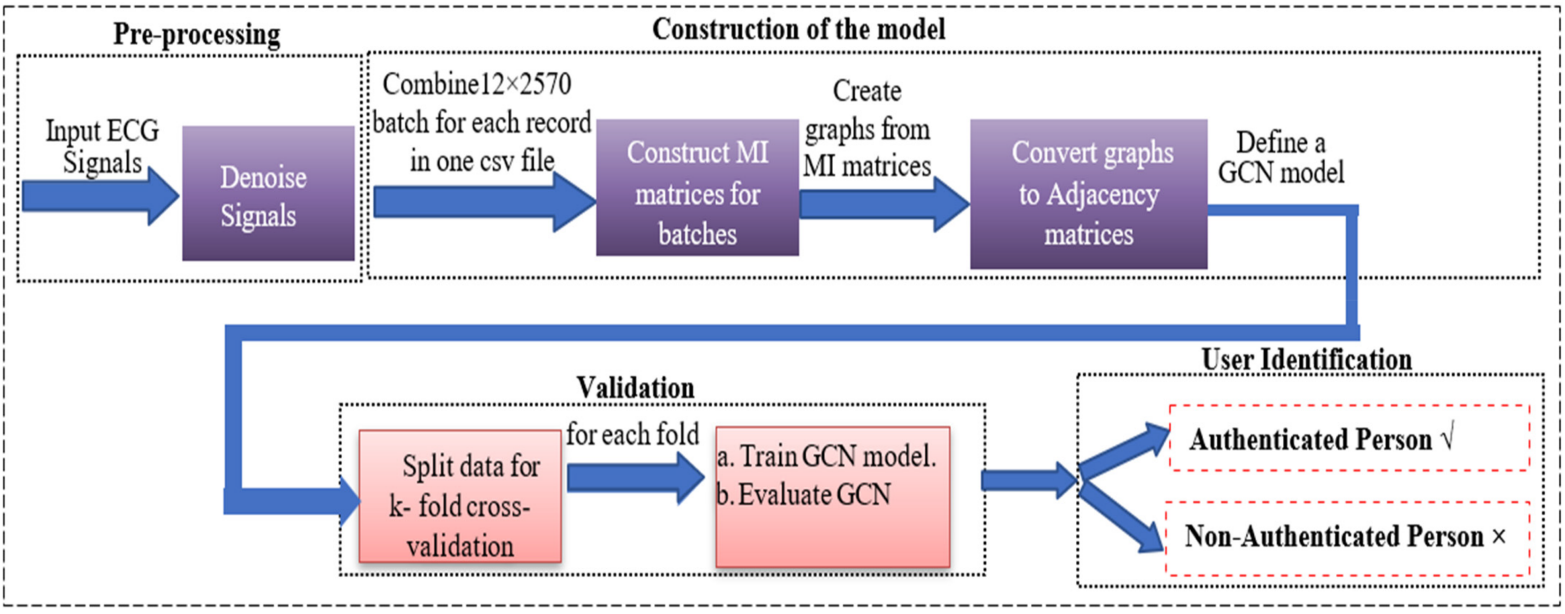
Block diagram for ECG-based 12-lead model for person identification using a GCN networks.

The proposed framework for human authentication leverages the use of Graph Convolutional Networks (GCNs) built on the Mutual Information (MI) of 12-lead ECG, along with advanced machine learning techniques, to model and analyze multi-lead ECG signals, ensuring robust individual authentication. The methodology has been structured into three phases, as illustrated in Figure 2: **(1) Pre-processing of the data**; **(2) Construction, training, and tuning of the model**; and **(3) Model Validation**.

The study employed the St. Petersburg Institute of Cardiological Techniques'12-lead Arrhythmia database (https://physionet.org/content/incartdb/1.0.0), which comprises 75 ECG recordings from 32 individuals, each lasting 10 s and sampled at 257 Hz, which generated 462,600 data points per lead. The following is an illustration of the methodology phases:

### 3.1 Data pre-processing

**Figure 3:**
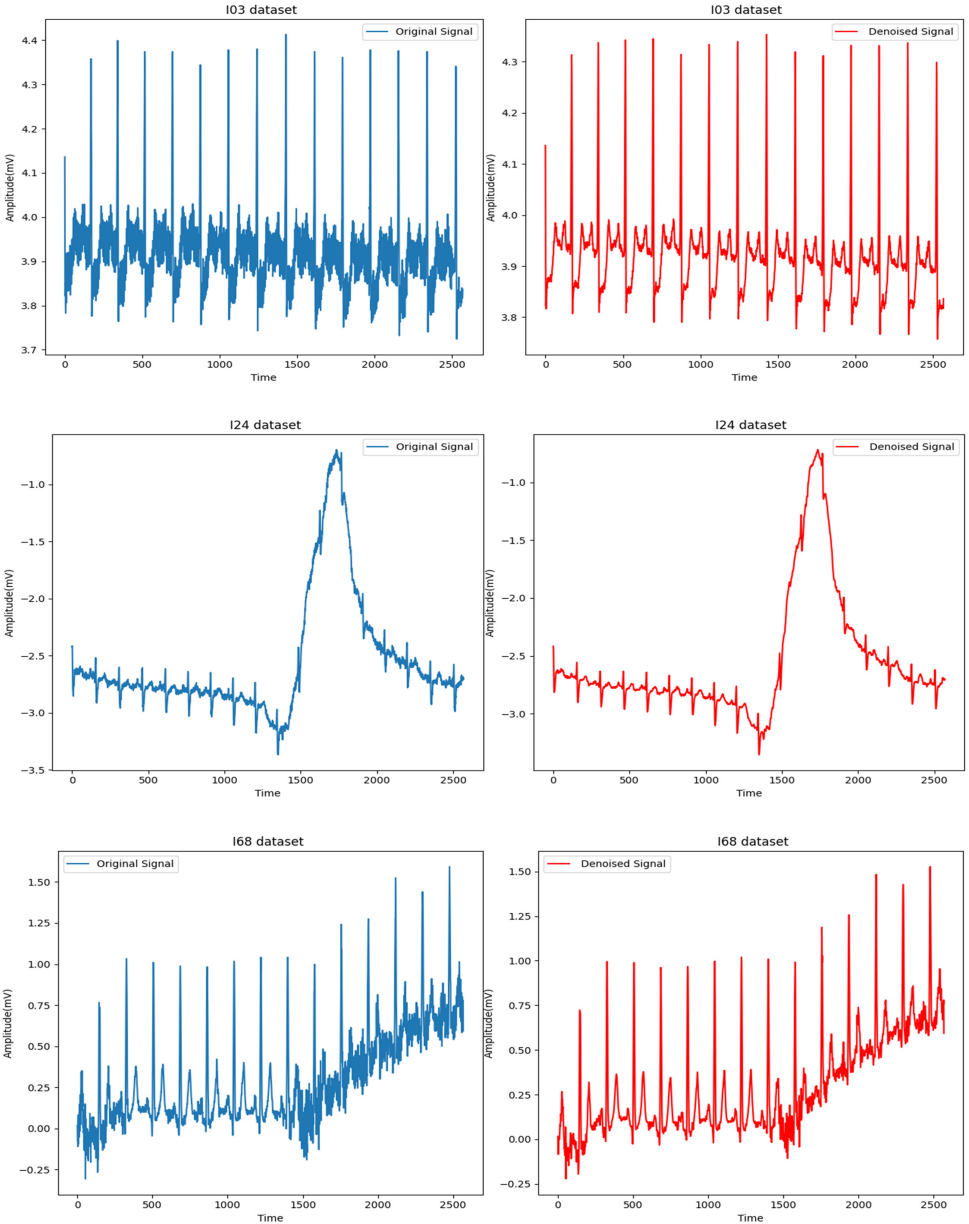
ECG time series for datasets (I03, I24, and I68) respectively before and after denoising.

Pre-processing is crucial for improving the quality of ECG signals by removing various types of noise that can obscure critical features necessary for human authentication. ECG signals are prone to several noise sources, including power line interference, motion artifacts, electrode contact noise, baseline wandering, and muscle contractions (39). The goal is to ensure that the pre-processed data is high quality and used effectively in downstream modeling. We implemented the sequential denoising technique proposed by Zheng et al. (39) To eliminate the following noise from the raw ECG data:

1.1. Power Line Interference (PLI): PLI is a familiar noise artifact in biomedical signals resulting from 50/60 Hz interference from electrical sources. This type of noise is particularly problematic for ECG signals, which typically lie in the 0.5 Hz to 50 Hz range (40). A Butterworth low-pass filter was applied to remove high-frequency noise above 50 Hz, preserving the relevant ECG signal while effectively reducing PLI.
1.2. Baseline Wandering: Baseline wandering arises from slow, Low-frequency drifts caused by respiration or subject movement. This drift can obscure important ECG features, notably the P, QRS, and T waves. A locally estimated scatterplot smoothing (LOESS) filter addresses this. LOESS adapts to the local trends of the data, ensuring that baseline drift is smoothed out without distorting the essential waveform characteristics (39).
1.3. Motion Artifacts and Muscle Noise: Motion artifacts result from subject movements, while muscle contractions introduce high-frequency disturbances into the ECG signal. A Non-Local Means (NLM) denoising algorithm was used to suppress these types of noise. NLM works by averaging similar patterns within the signal, making it particularly effective for preserving ECG waveform details while eliminating spurious noise (39). Results (see Figure 3) demonstrate the effectiveness of the pre-processing methods on the I03, I24, and I68 datasets, respectively. The signal in blue indicates considerable noise in the raw ECG time series. After denoising, the ECG signals become more straightforward to interpret and more suitable for further modeling, as evident in the corresponding red signals in the figure.

### 3.2 Model construction, training, and tuning

Following data cleaning, we build a graph-based model of the ECG signals and train a Graph Convolutional Network (GCN) to identify and authenticate individuals. GCNs, or graph convolutional networks, are among the most popular and effective varieties of neural networks, also known as graph neural networks. Graph Neural Networks (GNNs), specifically designed for graph-based data, were first introduced in 2009 (41).

Graph data structures are composed of entities called nodes (or vertices) and relationships between them, known as edges. Unlike grid-like data (such as photos, where pixels have a defined two-dimensional structure), graphs offer a flexible framework that enables the encoding of complex relationships and data points. GNNs' versatility makes them perfect for domains where connections between entities are essential, such as knowledge graphs, biological networks, social networks, and recommendation systems (42).

GCNs expand the fundamental convolution mechanism of Convolutional Neural Networks (CNNs) to graph data. When convolution operations are performed on fixed grid structures, such as images, conventional CNNs assume grid consistency and spatial locality. GCNs adapt convolutional processes to graph designs, whereby nodes, representing discrete entities or data points, and edges, representing links between nodes, do not follow a predetermined grid structure and may have varying connectivity (43). This modification enables GCNs to handle non-Euclidean data, making them suitable for a wide range of applications. The primary objective of the convolution operation in Graph Convolutional Networks (GCNs) is to capture local neighborhood information surrounding each node in the graph. This successfully creates a feature representation that includes the node's properties and the information from its neighbors.

**Figure 4:**
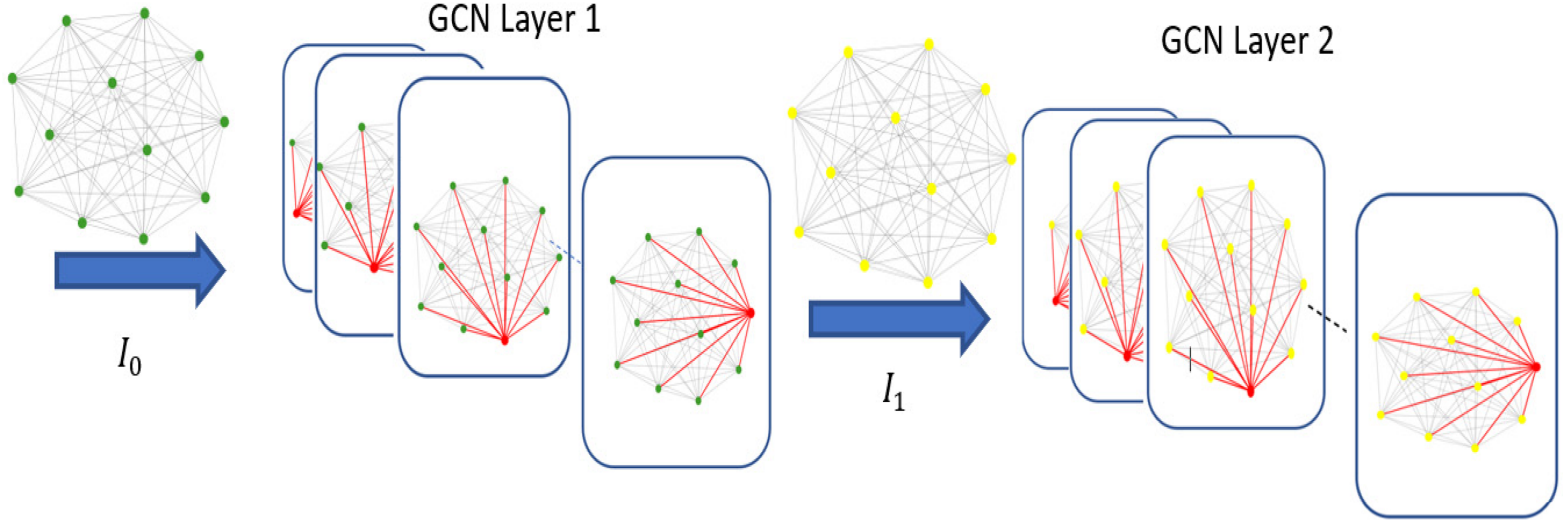
Neighbor aggregation process of GCN.

This iterative procedure, called neighbor aggregation or message passing (Figure 4), builds a multi-hop neighborhood representation that captures progressively more contextual information with each layer. It achieves this by having each node collect information from its immediate neighbors in one layer and then from the neighbors of neighbors in later layers.

The formal representation of a graph is G = (V, A). Individuals in a social network, molecules in a chemical complex, or medical symptoms in a network can all be represented by a set of nodes (or vertices) denoted as V. Depending on the context, a feature vector that describes each node is often associated with it. A *N* × *f* matrix. Where *N* is the number of nodes and *f* is the number of features, can be used to represent this (43).

Each row represents a node in the matrix, and each column represents a feature. A is the adjacency matrix, an *N* × *N* matrix that encodes node connections. The strength of the link is indicated by the weight of any edge held by A (i, j) between nodes I and J. A (i, j) = 0 without an edge. Both weighted graphs, in which the edges have varying strengths or significance, and unweighted graphs, in which the edges are either present or absent, can be represented by this matrix. A key component of GCNs is the adjacency matrix A, which specifies the connections within the network and determines how information should be transferred between nodes during the convolution process (42). For example, A would record friendships, track relationships, or engage with others in a social network, enabling the GCN to disseminate information about individuals via their social ties.

GCNs have many significant applications. They have been extensively utilized in domains including:

A. Social Network Analysis: GCNs can discover influential nodes or forecast relationships in networks like Facebook or Twitter.
B. Biological networks: GCNs facilitate the analysis of intricate biological networks, including disease-gene correlations or protein-protein interactions. This enables the classification of different types of diseases and the prediction of likely disease pathways.
C. Recommendation Systems**:** GCNs model user-item interactions by better understanding user preferences and item relationships.

The convolutional technique is extended from grid-like data (such as pictures) to irregular, structured data (graphs) by graph convolution. Nodes in a graph can have different numbers of neighbors and an irregular arrangement, unlike standard grids, where each pixel has a defined position and neighbors. To conduct convolution on graph data, a GCN must compile data from the node and its linked neighbors, representing the node's local neighborhood. The convolution operation on graphs is typically achieved through a polynomial filter based on the adjacency matrix **A**, capturing information from a node's neighborhood up to a certain distance. The following is the filtering function represented as a polynomial of the adjacency matrix A (see Table 2 for definitions of variables in Equation 1):

(1) $$H=h_{0}I+h_{1}A+h_{2}A^{2}+h_{3}A^{3}+\ldots +h_{k}A^{k}$$

After convolution, this formula applies a filter matrix H to the vertices (nodes) to create new node representations. The identity matrix, denoted as I, represents self-connections; each node considers its attributes.

**Table 2:** Definitions of variables in Equation (1).

Symbol	Description	Type
H	Filter matrix that results from the convolution operation.	*N* × *N* matrix
$h_{0},\;h_{1},\;\ldots ,\;h_{k}$	Scalar coefficients that control the participation of neighbors of a vertex in the convolution operation.	Scalars
I	Identity matrix, representing the vertex itself without propagation.	*N* × *N* matrix
A	Proximity (adjacency) matrix representing connections between vertices in a graph.	*N* × *N* matrix
$A^{i}$	i-th power of the adjacency matrix A, representing the number of steps of a vertex.	*N* × *N* matrix
*k*	Degree of the polynomial filter, determining the maximum number of steps for neighborhood propagation	Positive integer
*N*	Number of vertices in the graph (dimension of the adjacency matrix and filter matrix).	Integer

The scalar coefficients $h_{i}$ regulate the impact of neighbors at various hops or distances. For instance, $h_{1}$ controls the neighbors immediately adjacent to it, $h_{2}$ controls the neighbors two steps away, and so forth. In the graph, each node can incorporate information from neighbors k steps away by the adjacency matrix $A^{k}$, which is raised to the power of k (42). This polynomial filter defines a weighted combination of adjacency matrix powers, where each power corresponds to a specific distance from the node. For example:

*A*: Immediate neighbors (1-hop neighbors)
$A^{2}$: 2-hop neighbors (neighbors of neighbors)
$\;A^{3}$: 3-hop neighbors, and so on.

By combining information from these different distances, the GCN creates a multi-scale representation of each node, capturing local and global graph structures. After constructing the filter matrix H, it is applied to an input vertex matrix $V_{\mathrm{in}}$ (the initial feature representation of the nodes) to produce an output matrix $V_{\mathrm{out}}$, as follows (see Table 3 for definitions of variables in Equation 2):

(2) $$V_{out}=HV_{in}$$

This matrix multiplication combines node features based on the graph structure, allowing each node to have a representation that reflects its features and those of its connected neighbors. In practical terms, this operation enables each node to “learn” from its surroundings, making GCNs powerful tools for tasks that require a contextual understanding of relationships within the data (41).

**Table 3:** Definitions of variables in Equation (2).

Symbol	Description	Type
$V_{\mathrm{in}}$	Input vertex matrix (initial feature representation of nodes). Each row corresponds to a node's features.	$N\times F_{\mathrm{in}}$ matrix
H	The filter matrix is constructed from the graph structure [as defined in Equation (1)].	Scalars
$V_{\mathrm{out}}$	Output vertex matrix (updated feature representation of nodes after filtering).	$N\times F_{\mathrm{out}}$ matrix
*N*	Number of nodes in the graph (i.e., vertices).	Integer
$F_{\mathrm{in}}$	Number of input features for each node.	Integer
$F_{\mathrm{out}}$	Number of output features for each node (same as $F_{\mathrm{in}}$ in this case).	Integer

The critical innovation in our model is the use of mutual information (MI) to capture dependencies between different leads formed by the 12-lead ECG data, enabling the construction of an adjacency matrix used in graph convolutional neural network (GCN) training.

**Mutual Information:** Mutual Information (MI) serves as the basis for understanding the connections between multiple ECG leads. The notion of entropy is where Mutual Information (MI) first appeared. Uncertainty regarding the combination of two random variables (X, Y) is expressed in (3) by their joint entropy (41).

(3) $$H[\text{X},Y]=-\sum_{x\in X,y\in Y}\text{Pr}[X=x,Y=y]\cdot \text{log}\text{Pr}[X=x,Y=y]$$

where:

• **H[X, Y]:** The joint entropy, measuring the uncertainty or information contained in the joint distribution of X and Y.
• **ΣΣ:** Summation over all possible values of *X* and *Y*.
• **Pr[*X*** **=** ***x*, *Y*** **=** ***y*]:** The joint probability of *X* = *x* and *Y* = *y*.
• **Log: The logarithm, typically based on** 2 in information theory.

Uncertainty of a random variable (*X*) that continues after (*Y*) is known is expressed in (4) by its conditional entropy concerning *Y*:

(4) $$H[\text{X}|\text{Y}]=-\underset{x\in X,y\in Y}{\sum }\text{Pr}[X=x,Y=y]\cdot \text{log}\text{Pr}[X=x|Y=y]$$

where:

• **H[X|Y]:** The conditional entropy that tells us the remaining uncertainty in X, given that we already know Y.
Pr[X=x|Y=y]**:** The Conditional probability of X = x given Y = y.

#### 3.2.1 Model construction

**To construct the GCN-MI model**, we first compute the Mutual Information (MI) matrix from ECG signals, which is then transformed into the adjacency matrix used by the Graph Convolutional Network (GCN). The step-by-step process is as follows:

##### 3.2.1.1 ECG signal preprocessing

• The raw ECG signals undergo bandpass filtering (0.5–40 Hz) to remove noise.
• Each ECG segment is normalized using Z-score normalization to ensure sample consistency.
• Feature extraction is performed using wavelet transforms to capture time-frequency representations.

##### 3.2.1.2 Computation of the MI matrix

An MI measures the information shared by two random variables, such as the signals from two leads. It quantifies the information one lead provides about another and records linear and non-linear correlations. The formula (5) is used to determine the MI between two ECG leads, X and Y (see Table 4 for definitions of variables in Equation 5):

(5) $$I\left(X;\text{Y}\right)=\underset{x\in X,y\in Y}{\sum }\text{Pr}\left[X=x,=y\right]\cdot \text{log}\left(\frac{\text{Pr}\left[X=x,Y=y\right]}{\text{Pr}\left[X=x]\cdot \text{Pr}[Y=y\right]}\right)$$

**Figure 5:**
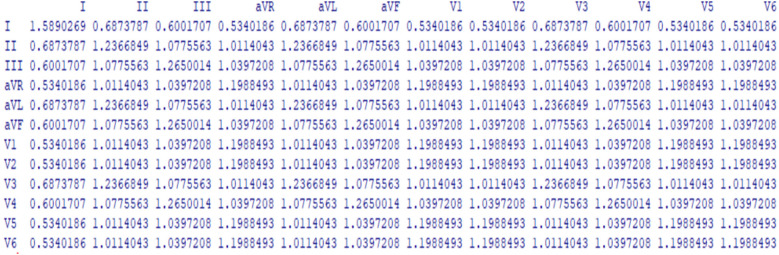
A single individual's cardiac lead mutual information displayed in a 12 × 12 matrix.

This computation yields an MI matrix (Figure 5) that provides a pairwise representation of the inter-lead relationships.

**Table 4:** Definitions of variables in Equation (5).

Symbol	Description
I (X; Y)	Mutual information represents the amount of information shared between X and Y.
$\;\sum_{x\in X,y\in Y}$	Summation over all possible values of X and Y.
Pr [X = x, Y = y]	The joint probability of X = x and Y = y.
log	Logarithmic function (typically base 2), measuring the amount of information in bits.
Pr[X = x]·Pr[Y = y]	Product of marginal probabilities of X = x and Y = y.
$\;\frac{\mathrm{Pr}[X=x,Y=y]}{\text{Pr}[X=x]\cdot \text{Pr}[Y=y]}$	Pointwise mutual information, comparing the joint probability with the product of marginals.

Each element in this matrix quantifies the information shared between a pair of leads, forming the foundation for the adjacency matrix in the GCN.

##### 3.2.1.3 Conversion of the MI matrix into an adjacency matrix

• A threshold *τ* is applied to remove weak connections (see Equation 6):
  (6) $$A_{ij}=\{\begin{array}{cc} M_{ij}, & \mathrm{if}\;M_{ij}>\tau \\ 0, & \mathrm{otherwise} \end{array}$$
• The adjacency matrix A is symmetrically normalized using:
  (7) $$\hat{A}=D^{-1/2}AD^{-1/2}$$
  where:
**A:** Represents the connections between nodes in a graph.
**D:** is the degree matrix of A.
$D^{\frac{-1}{2}}$, Which scales each node's connections based on its degree.

##### 3.2.1.4 Graph convolutional network (GCN) construction

The GCN model includes:

• Nodes represent ECG feature vectors from different time steps.
• Edges are established based on the MI matrix thresholding.
• Three graph convolutional layers, each using a ReLU activation function.
• A dropout layer (*p* = 0.5*p* = 0.5*p* = 0.5) is applied to prevent overfitting.
• The final layer outputs class probabilities
• using **SoftMax activation**.
• The model is trained using the **Adam optimizer** with a learning rate of 0.001, and the loss is computed using **categorical cross-entropy**.

**Figure 6:**
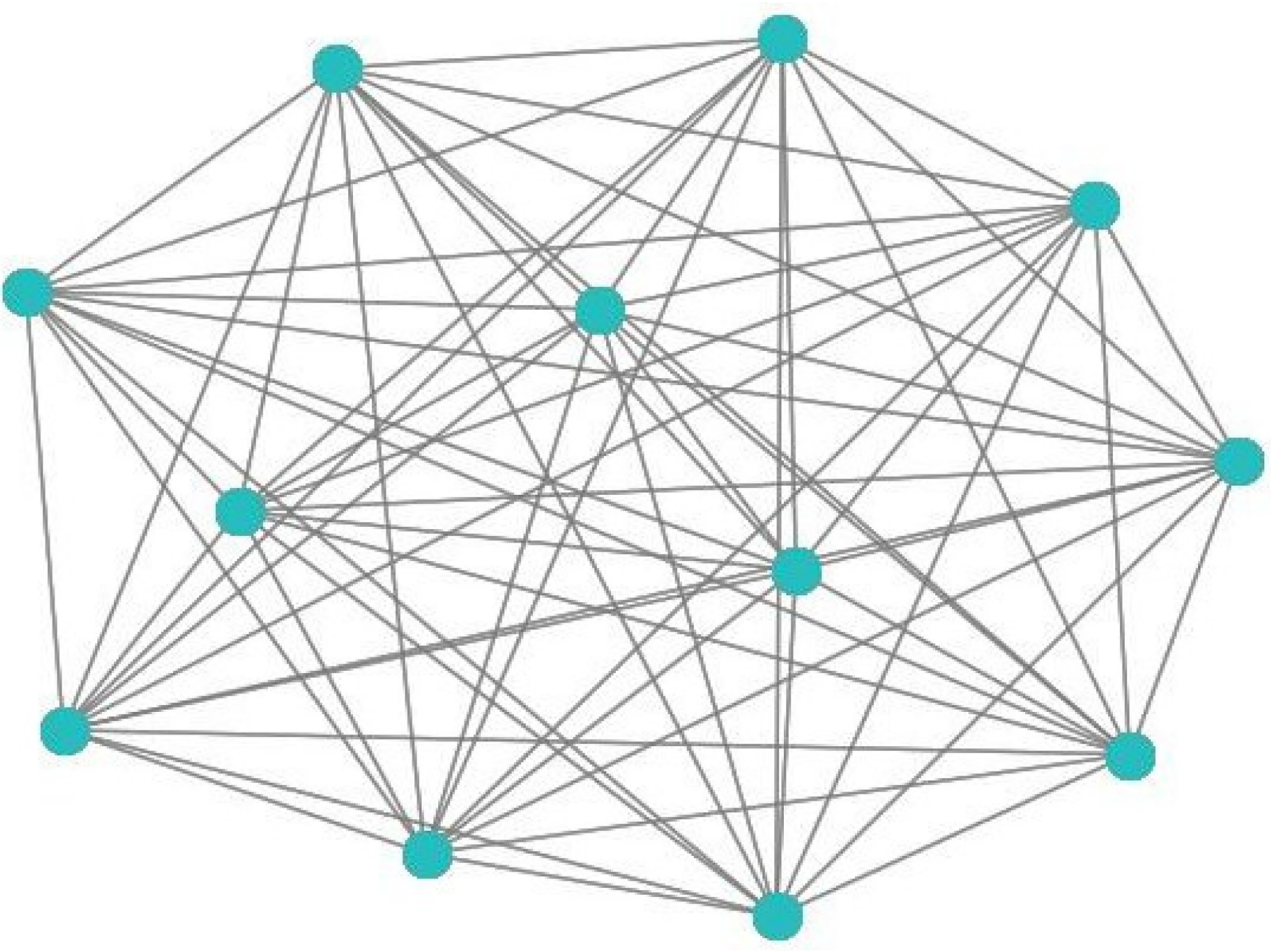
12-lead ECG-based graphs constructed from MI matrices.

This process's pseudocode is described in Algorithm 1, and Figure 6 shows an instance of a built graph via connection (MI) between the 12 leads.

The GCN-MI model consists of multiple graph convolutional layers that process ECG signals structured as a graph, where edges are defined by Mutual Information (MI) relationships. The overall structure is outlined below:

(1) **Input Layer:** In this layer, A matrix consisting of 75 batches belonging to 75 persons each of (2,570 × 12) rows (where 12 represents the ECG leads, and 2,570 is the number of extracted features per lead) and an adjacency matrix (12 × 12 represented relationships between ECG leads based on mutual information (MI), was fed into the GCN as the input, where each entry (i,j) corresponds to the MI value quantifying the dependency between lead i and lead j in the 12 × 12 adjacency matrix, this matrix serve as the GCN's structural backbone, encapsulating the underlying dependencies and correlations between ECG leads. This study generated the adjacency matrix via the R program and utilized MI values to define graph edges that capture intricate relationships between cardiac leads. An R program is a script or set of instructions written in the R programming language, a popular open-source language and environment specifically designed for statistical computing, data analysis, and visualization. In our study, we constructed the first layer as a 2D convolutional layer with 64 filters, a kernel size of (1, 1), strides of (1, 1), and no padding (valid). Then, we used the ReLU activation function described in (9) to extract low-level features from the graph.

**Preprocessing:** Each ECG feature undergoes *Z* score normalization before entering the GCN

**Algorithm 1:** GCN-MI model construction

1. **ECG Signal Preprocessing:** • Apply bandpass filtering (0.5–40 Hz) to remove noise. • Normalize each ECG segment using Z-score normalization. • Extract features using wavelet transforms for time-frequency representation. 2. **Computation of the MI Matrix:** • MI quantifies shared information between ECG leads, capturing linear and non-linear correlations. • Compute MI using: $I\left(\text{X};\text{Y}\right)=\underset{x\in X,y\in Y}{\sum }\mathrm{Pr}\left[X=x,=y\right].\log\left(\frac{\mathrm{Pr}\left[X=x,Y=y\right]}{\mathrm{Pr}\left[X=x].\text{Pr}[Y=y\right]}\right)$ • Construct an **MI matrix**, where each entry represents MI between two ECG leads. 3. **Conversion of MI Matrix into an Adjacency Matrix:** • Apply threshold ***τ*** to remove weak connections: $$A_{ij}\;=\{\begin{array}{cc} M_{ij}, & \mathrm{if}\;M_{ij}>\tau \\ 0 & \mathrm{otherwise} \end{array}$$ • Normalize adjacency matrix using: $\hat{A}=D^{-1/2}AD^{-1/2}$ Where **D** is the degree matrix. 4. **Graph Convolutional Network (GCN) Construction:** • Nodes represent ECG feature vectors from different time steps. • Edges are created based on MI thresholding. • Use three graph convolutional layers with ReLU activation. • Apply dropout layer (***p* =0.5**) to prevent overfitting. • Final layer outputs class probabilities using SoftMax activation. • Train model using Adam optimizer (**learning rate = 0.001**) and categorical cross-entropy loss.

(2) **Graph Convolutional Layers:** Convolutional layers are critical in GCN, especially in image and signal processing tasks. In our study, multiple layers were used to extract features from ECG data, enabling the model to learn discriminative patterns associated with individuals' unique heart rhythms. The convolution operation is defined as:
  (8) $$H^{\left(l+1\right)}=\sigma (D^{-1/2}A{\text{D}}^{-1/2}H^{\left(l\right)}W^{\left(l\right)})$$
  where:
- A is the adjacency matrix,
- D is the degree matrix,
- *H^(l)^* is the node feature matrix at layer *l*,
- *W^(l)^* are the learnable weights and
- *σ* is the activation function.

A sequence of graph convolutional layers progressively enhances the network's ability to recognize complex patterns and relationships between ECG leads. In each layer, the GCN processes information from neighboring nodes and produces new feature representations by weighing the connections in the graph. The model aggregates more global patterns with each successive layer, helping it understand local dependencies (from neighboring nodes) and global dependencies (across more distant nodes). The output of each convolution layer is given to the next layer. As (44) described, including two to three convolutional layers can help capture deeper GCN insights. In our study, the output of the convolutional layer was flattened and passed through 13 dense layers, each consisting of 64 neurons, and the ReLU activation function (7) was used to learn higher-level representations.

(3) **Activation Functions:** Each graph convolutional layer is followed by a ReLU activation function to introduce non-linearity, which is crucial for learning complex, non-linear patterns in ECG data.

ReLU activation is defined as:

(9) f(x)=max(0,x)

If the input *x* > 0, the output is *x* itself.
If the input *x* ≤ 0, the output is 0.

where:

*x* refers to the raw output from the graph convolutional layer before applying the ReLU activation. The ReLU activation function lets the GCN on significant signal variations by zeroing out non-informative features (negative values). This helps the model differentiate between subtle, nuanced relationships and identify meaningful patterns that distinguish authentic and non-authentic samples.

(4) **Output Layer:** The final output layer is a single dense neuron with a sigmoid activation function (8), mapping the output to a probability range of [0, 1] for binary classification. In the proposed method, the output layer of the GCN was designed to perform the final classification task by converting extracted features into a probabilistic score, determining the likelihood that an ECG sample corresponds to a particular individual. It received input from the final graph convolutional layer, which produces high-level feature representations of the graph, with nodes representing the 12 ECG leads and edges weighted by mutual information (MI) values.

The layer consists of a trainable weight matrix that maps the feature dimensions to the output space, and a sigmoid activation function was applied to generate a probability score between 0 and 1. The sigmoid function is defined as in Equation 10:

(10) $$\sigma (x)=\;\frac{1}{1+e^{-x}}$$

where:

• *x* is the input to the function.
• *e* is the base of the natural logarithm (approximately 2.718).

#### 3.2.2 Model training and tuning

The GCN-MI model's training process follows a supervised learning approach. The model learns from labeled ECG feature matrices derived from the 12-lead ECG signals according to the following specifications:

• **Dataset:** The model was trained using the St. Petersburg Institute of Cardiological Techniques 12-lead Arrhythmia Database, which contains 75 ECG recordings from 32 individuals, each sampled at 257 Hz.
• **Input Representation:** Each ECG recording was transformed into a feature matrix of size (12 × 2,570), with 12 leads as graph nodes and an adjacency matrix derived from MI computations.
• **Optimization Algorithm:** The model was trained using the Adam (Adaptive Moment Estimation) optimizer, which was chosen because:
  • It adapts learning rates for each parameter dynamically.
  • It effectively handles sparse gradients, making it suitable for graph-based data.
• **Loss Function**: The Binary Cross-Entropy (BCE) loss function optimized the model. BCE is a commonly used loss function for binary classification problems, ensuring that the predicted probabilities match the accurate class labels.

The BCE loss function is defined as in Equation 11:

(11) $$L=\frac{-1}{N}\overset{N}{\underset{i=1}{\sum }}[y_{i}log(p_{i})+(1-y_{i})\text{log}(1-p_{i})]$$

where:

• *N* is the number of samples.
• *y_i_* is the ground truth label.
• *p_i_* is the predicted probability for sample *i*.

Table 5 shows the hyperparameters tuned through experimental validation to achieve optimal model convergence.

**Table 5:** The parameters of the GCN network with 13 hidden convolution layers and the MI adjacency matrix.

Parameter	Value
Epochs	600
Learning Rate	0.9889
Hidden Layers	13 with 64 neurons each
Dropout	0.2

##### 3.2.2.1 Model validation

We implemented a rigorous validation strategy to evaluate the proposed GCN-MI model's performance and generalization capability. This process ensures that the model is optimized for the training dataset and maintains high accuracy on unseen data.

A. **Dataset Splitting Strategy:**

The dataset was randomly split into 70% training, 15% validation, and 15% test sets. The validation set was used for hyperparameter tuning, while the test set provided an unbiased assessment of the model's ability to generalize.

B. **Leave-One-Out Cross-Validation (LOO-CV):**
  • Each ECG sample is used as a test set once, while the remaining samples are used for training.
  • Ensures the model is tested on all data points without data leakage.

C. **K-Fold Cross-Validation:**
  • The dataset was split into *K* = {60, 65, 70, 75} folds.
  • Each fold is used as a test set, while the remaining (K − 1) folds are used for training.
  • Helps assess the model's generalization performance across different subsets of data.
  • Ensuring that a particular dataset partitioning did not bias results.

D. **Evaluation Metrics:**

The model's performance was assessed using multiple classification metrics, including:

1. **Accuracy:** Overall classification correctness.
2. **Precision, Recall, and F1-score:** Evaluating the ability to classify individual ECG signals correctly.
3. **Area Under the ROC Curve (AUC):** Measures the model's ability to distinguish between users.

E. **Equal Error Rate (EER):** is the error value when the false rejection rate and the false acceptance rate are equal.
F. **Statistical Robustness and Significance Testing:**

We computed 95% confidence intervals (CIs) for accuracy and F1-score to confirm the statistical significance of the model's results. Additionally, we performed paired t-tests between different k-fold validation runs to verify the reliability of the reported improvements.

G. **Impact of Model Complexity on Generalization:**

As detailed in the Results section, we analyzed the impact of varying the number of layers (5, 10, 15) in the GCN-MI model. We examined the performance across different k-fold values (60, 65, 70, 75), confirming that the model maintains high accuracy across various configurations.

While these validation methods provide strong evidence of the model's robustness, explicit cross-subject prediction—where the model is trained on one set of individuals and tested on an entirely different set—was not separately evaluated in this study. However, given that LOO-CV ensures that each sample is excluded from training at least once, it inherently simulates cross-subject scenarios to some extent. Future research could further strengthen the assessment by employing a leave-one-subject-out (LOSO) validation, where all individuals are excluded during training to test true cross-subject generalization. Despite this, our results indicate that the GCN-MI model can accurately distinguish subjects across multiple data splits, reinforcing its potential for real-world biometric authentication.

**Figure 7:**
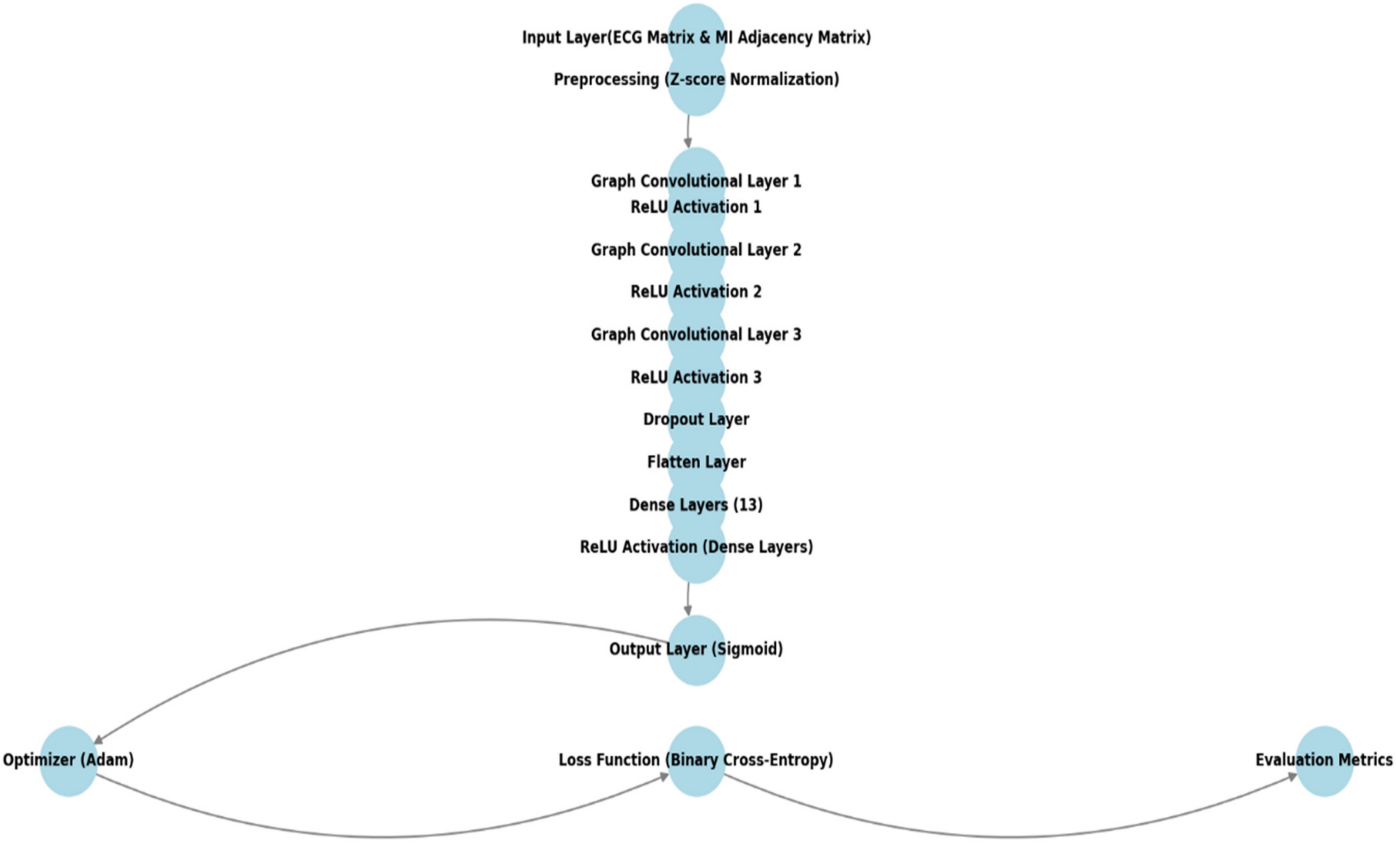
Architecture of the proposed GCN-MI model.

**Proposed model's architecture drawing:** We present a drawing of the proposed model's architecture, which effectively handles user authentication using Electrocardiogram (ECG) signals by leveraging Graph Convolutional Networks (GCNs) and deep learning techniques, as shown in Figure 7.

## 4 Results

In this study, multiple graph convolutional layers, which are the core of the GCN model, were employed to aggregate information from neighboring nodes (leads) before and after noise suppression and progressively extract more features. The best network configurations were identified through experiments with various convolutional layers, specifically the GCN-MI-5, GCN-MI-10, and GCN-MI-15 models. The performance of the proposed model is shown in Table 6.

**Table 6:** Identification performance of the proposed model with 5, 10, and 15 layers before and after noise suppression.

	Accuracy (%)					
Fold	GCN-5 (Denoised)	GCN-10 (Denoised)	GCN-15 (Denoised)	GCN-5 (Noisy)	GCN-10 (Noisy)	GCN-15 (Noisy)
Fold 75	100	100	100	100	100	90.66
Fold 70	97.14	98.57	97.14	97.08	98.57	93.57
Fold 65	98.46	96.92	98.46	96.92	96.15	93.07
Fold 60	94.16	90	93.33	94.16	89.50	93.33

To evaluate the proposed model's effectiveness, we analyzed its accuracy, precision, recall, F1 score, ROC-AUC, and EER and compared performance across various models.

1. **Performance Metrics for Biometric Authentication:**

The formulas for quantifying measurements are listed below:

• Accuracy: a standard evaluation metric in classification tasks that measures the proportion of correctly classified instances out of the total instances. It is defined as in Equation 12:
  (12) $$\text{Accuracy}\;=\frac{TP+TN}{TP+TN+FP+FN}$$
• Precision (P): Measures how many predicted positive samples are positive. It is defined as in Equation 13:
  (13) $$\text{P}\;=\frac{TP}{TP+FP}\;$$
• Recall (Sensitivity) (R): Measures how well the model captures actual positives. It is defined as in Equation 14:
  (14) $$\text{R}\;=\frac{TP}{TP+FN}\;$$
• F1-score: The harmonic means of precision and recall. It is defined as in Equation 15:
  (15) $$\text{F}1\;=2\times \frac{P\times R}{P+R}\;$$
• **ROC Curve & AUC:** The Receiver Operating Characteristic (ROC) curve plots the True Positive Rate (TPR) vs. the False Positive Rate (FPR). The Area Under the Curve (AUC) summarizes classification ability.

Our findings confirm that noise suppression significantly enhances biometric authentication accuracy, thereby reducing the negative impact of power-line interference, motion artifacts, electrode contact noise, and baseline wandering. Table 6 presents the accuracy results for different models and *k*-fold settings before and after noise suppression. Table 6 shows that the GCN-5 and GCN-10 models achieve perfect stability at fold 75 and maintain relatively high performance at fold 70 across all conditions. This can be attributed to the increased availability of training data at higher folds, resulting in better generalization and model optimization. The GCN-15 model is significantly affected by noise, with accuracy decreasing from 100% to 90.66% at Fold-75 and from 97.14% to 93.57% at Fold-70. This suggests that deeper models, such as GCN-15, maybe more noise-sensitive due to their greater reliance on feature consistency across multiple layers. Our proposed model benefited from reducing variability and noise in the ECG data, which allowed even the most complex model (GCN-MI-15) to perform effectively.

The GCN-MI-5 denoised model appears more robust at fold 60, with a higher accuracy of 94.16% compared to GCN-MI-10 (90%) and GCN-MI-15 (93.3%). This suggests that simpler models (like GCN-MI-5) are better suited for scenarios with limited training data, as they are less prone to overfitting.

**Figure 8:**
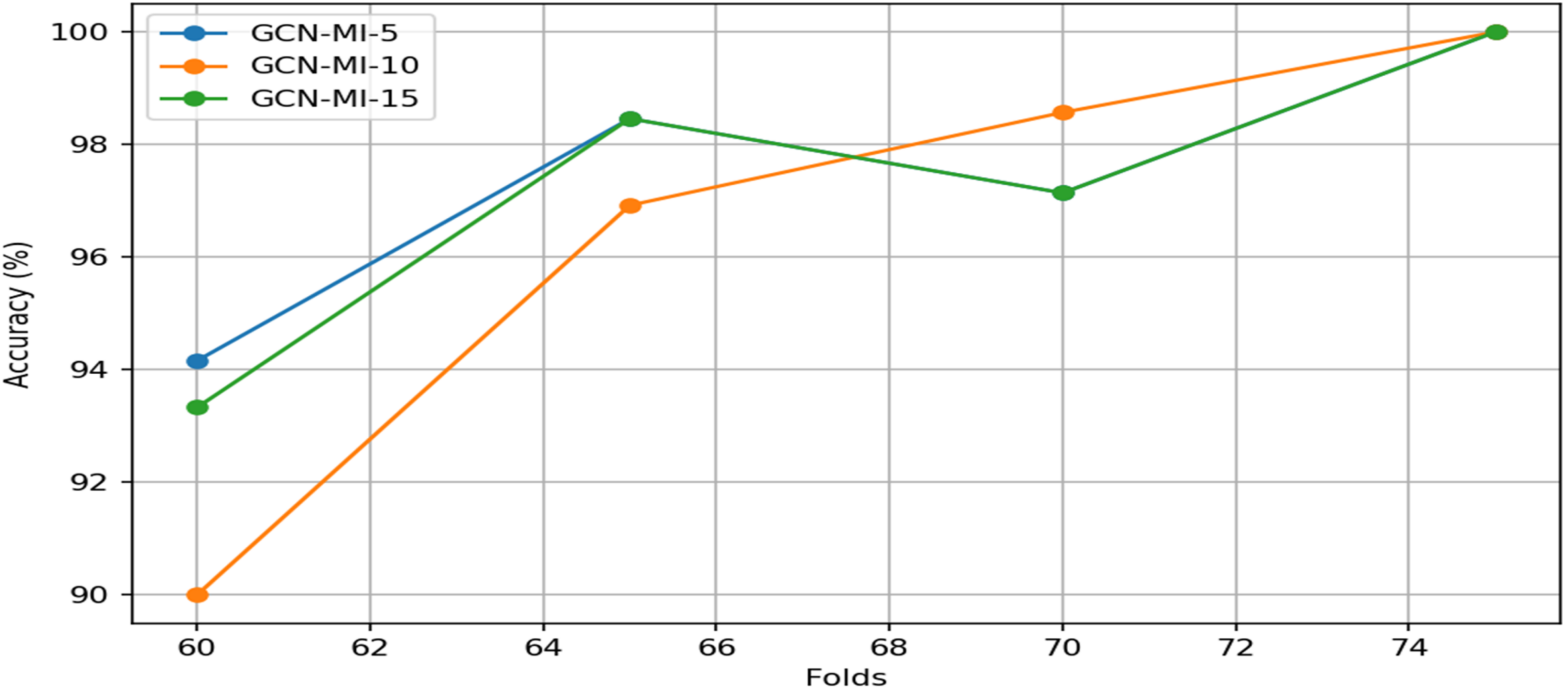
Authentication accuracy for ECG-based 12-lead GCN-MI model.

**Figure 9:**
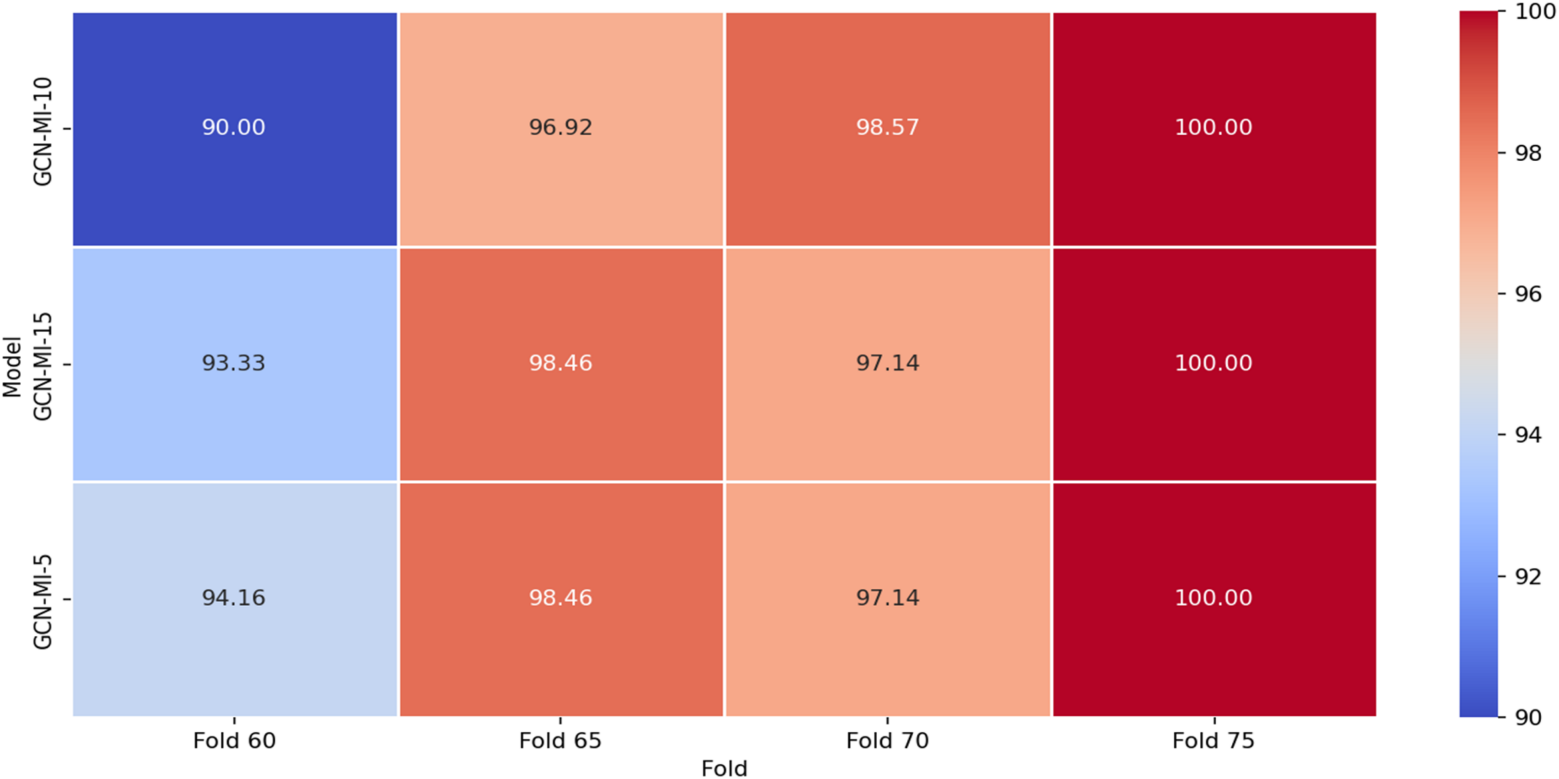
Accuracy heatmap for the proposed GCN-MI model.

The results in Table 6 also indicate that increasing the number of layers (from 5 to 15) does not necessarily guarantee better performance; deeper models (GCN-MI-10, GCN-MI-15) require more data to leverage their complexity, while simpler models (GCN-MI-5) are more efficient and less computationally expensive, making them ideal for real-time applications or resource-constrained environments. The differences in accuracy across folds highlight the importance of diverse and representative datasets. The visualization of how the authentication accuracy values change across the different *K*-folds in different layers (5, 10, 15) is shown in Figure 8. The *X*-axis represents the various values of folds (60, 65, 70, 75), and the *Y*-axis represents the accuracy value in each layer. Each line corresponds to a different model (GCN-MI-5, GCN-MI-10, GCN-MI-15), showing how each model's accuracy fluctuates as the number of folds changes. The chart highlights the differences between the models. It demonstrates the variability in their performance as K changes, clearly showing the advantages of using the GCN-MI approach for ECG-based authentication. The Heatmap in Figure 9 illustrates the model's authentication effectiveness and highlights its high classification accuracy.

The results demonstrate that the GCN-MI models are particularly effective for ECG-based authentication, which involves recognizing individuals based on their unique ECG signals. The GCN-MI models can capture complex and subtle patterns in the ECG data, which is critical authentication, for slight variations in ECG signals can distinguish individuals.

2. **Performance Comparison Across Different Models**

This study illustrates the benefits of integrating intricate data linkages into biometric authentication systems. The GCN-MI technique, which focuses on the complex relationships present in 12-lead ECG data, represents a breakthrough in biometric identification, offering a more accurate and inherently safer solution. This opens up new possibilities for creating advanced biometric systems that can be applied in various real-world scenarios, improving security and reliability across numerous industries and applications. Table 7 comprehensively evaluates model performance through additional evaluation metrics. Furthermore, as cybersecurity threats evolve.

**Table 7:** Evaluation of the proposed model's performance using accuracy, precision, recall, F1-score, AUC-ROC, and EER.

Model	Folds	Accuracy	Precision	Recall	F1-Score	AUC-ROC	EER
GCN-MI-5	Fold 60	94.16%	94.74%	94.74%	94.74%	0.95	0.05
	Fold 65	98.46%	97.37%	97.37%	97.37%	0.95	0.05
	Fold 70	97.14%	97.37%	97.30%	97.37%	0.95	0.05
	Fold 75	100%	100%	100%	100%	1	0.01
GCN-MI-10	Fold 60	90.00%	91.89%	89.47%	90.67%	0.95	0.05
	Fold 65	96.92%	97.37%	97.37%	94.74%	0.95	0.05
	Fold 70	98.57%	98.67%	97.37%	98.04%	0.95	0.05
	Fold 75	100%	100%	100%	100%	1	0.01
GCN-MI-15	Fold 60	93.33%	94.59%	92.11%	93.33%	0.95	0.05
	Fold 65	98.46%	97.37%	97.37%	97.37%	0.95	0.05
	Fold 70	97.14%	97.37%	97.37%	97.37%	0.95	0.05
	Fold 75	100%	100%	100%	100%	1	0.01

ECG-based biometrics may offer a robust defense against emerging attack vectors, such as AI-driven attempts to mimic other biometric patterns.

**Figure 10:**
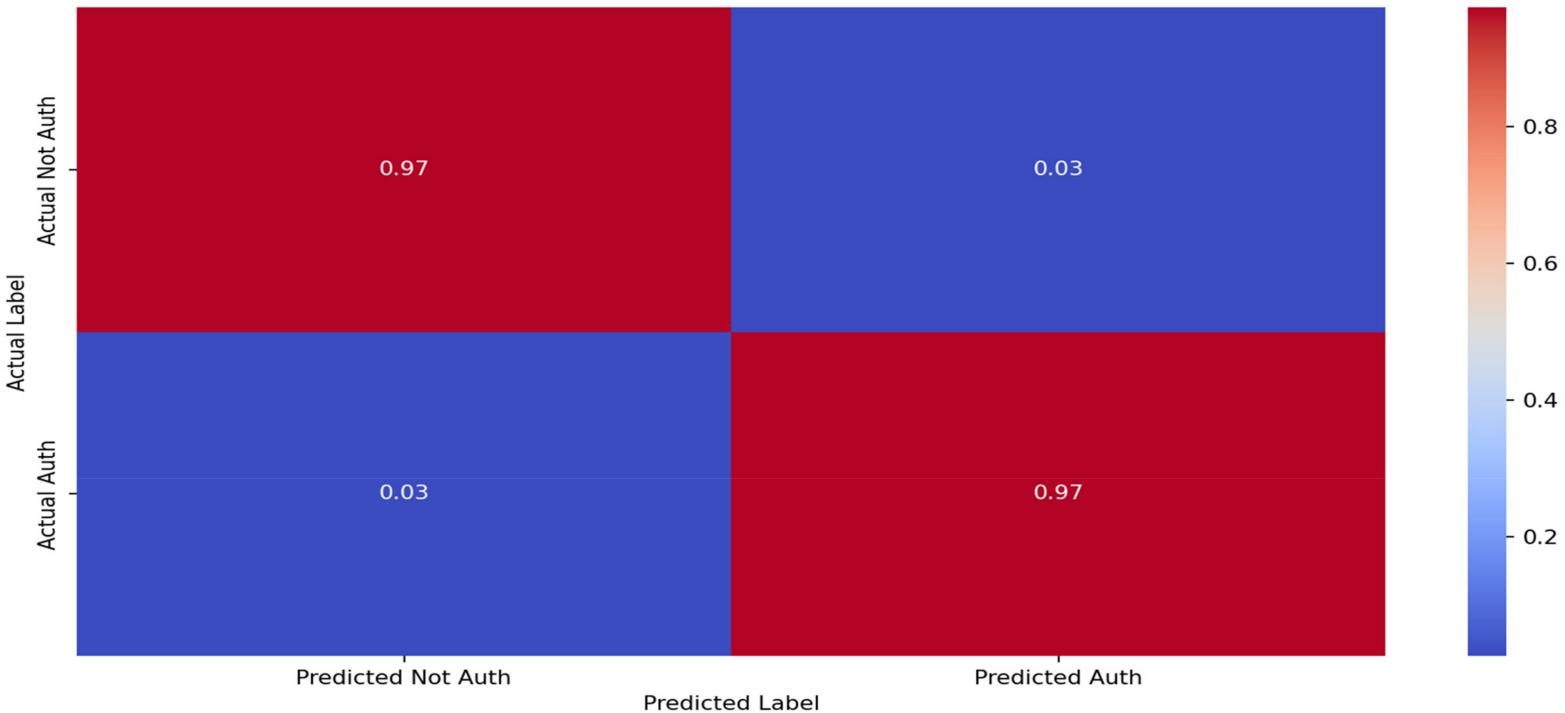
Confusion matrix for the GCN-MI-5 (fold 65) model.

The findings in this study underscore the feasibility of GCN-MI models for real-world, security-focused applications, where accuracy and adaptability are paramount. The confusion matrix in Figure 10 highlights the GCN-MI-5's ability to balance authentication false positives and negatives on 60 folds, which is crucial for biometric systems. This approach's implications are far-reaching.

By demonstrating that incorporating complex inter-lead relationships via MI can significantly improve the accuracy of biometric systems; the study paves the way to deploy more secure and reliable applications in critical sectors. For instance, in the financial industry, where secure user identification is paramount, ECG-based biometric authentication could provide additional protection against identity theft and fraud.

Similarly, ECG-based identification could be used in healthcare to securely verify patients, ensuring that medical records and treatment plans accurately match the right individual. ECG-based biometrics may offer a safer alternative to conventional authentication techniques in personal security, making it more challenging for unauthorized users to access secure systems or devices. For instance, in personal technology, ECG authentication could be applied to unlock smartphones, laptops, or IoT devices, providing a unique security layer inherent to the individual.

This method could also be integrated into wearable devices, allowing seamless yet secure access to restricted areas or sensitive information. Furthermore, as cybersecurity threats evolve, ECG-based biometrics may offer a robust defense against emerging attack vectors, such as AI-driven attempts to mimic other biometric patterns.

Overall, adopting ECG-based biometric systems, such as GCN-MI, is a digital approach to transforming identity verification across various high-stakes domains. It offers a security solution that is both highly personalized and resilient, meeting the rising demand for reliable authentication in an increasingly digital, interconnected world.

**Statistical Significance Analysis:** While all GCN-MI models achieved high accuracy, it is crucial to determine whether the performance differences between them are statistically significant.

To this end, Table 8 presents the 95% confidence intervals (CIs) for each model's accuracy and F1 score, indicating the range within which the accurate performance metrics are expected to lie with 95% confidence. Furthermore, we conducted paired t-tests across different k-fold settings (60, 65, 70, and 75) to assess whether the observed variations in accuracy are statistically significant; the results are shown in Table 9.

• **Null Hypothesis (H₀):** There is no significant difference in accuracy between different GCN-MI models.

**Table 8:** Compute confidence intervals for the proposed model.

Model	Accuracy 95% CI	F1-Score 95% CI
GCN-MI-5	95.2349, 99.2850	95.3975, 99.3425
GCN-MI-10	92.1425, 99.2850	92.5125, 99.5100
GCN-MI-15	94.2825, 99.2850	94.3399, 99.3425

**Table 9:** Statistical comparison of model performance (paired *t*-test results).

Comparison	*t*-statistic	*p*-value(*α* = 0.05)	Significance
GCN-MI-5 vs. GCN-MI-10	0.89	0.438	H_0_
GCN-MI-5 vs. GCN-MI-15	1.00	0.391	H_0_
GCN-MI-10 vs. GCN-MI-15	−0.84	0.463	H_0_

**Interpretation of Results:** The results of the paired t-tests are in Table 9. indicate that all *p*-values are greater than 0.05, suggesting that the observed differences in accuracy between the models are not statistically significant. While there are numerical variations in accuracy across different k-fold values, these differences do not provide sufficient statistical evidence to conclude that one model consistently outperforms the others. This suggests that the observed performance fluctuations may be attributed to sample variability rather than an inherent superiority of any specific model, emphasizing the need for further validation on more extensive and diverse datasets to confirm these findings.

## 5 Discussion

ECG authentication approaches and related techniques in biometric-based identity systems have rapidly emerged as a critical focus within security studies, driven by the need for highly secure and individualized authentication methods. Unlike traditional biometric approaches, ECG authentication leverages unique heart patterns resilient against physical duplications, offering promising applications for health information systems (HIS), secure banking, and personal device access. To enhance the reliability and accuracy of these systems, numerous studies have explored foundational and innovative solutions to address persistent challenges in ECG authentication.

In this section, we compared the performance of the proposed GCN-MI model against several of these state-of-the-art studies to show the significant advantages of the GCN-MI model against other models in terms of:

A. **Accuracy and Dataset Utilization:**
  1 **Superior Accuracy**

**Figure 11:**
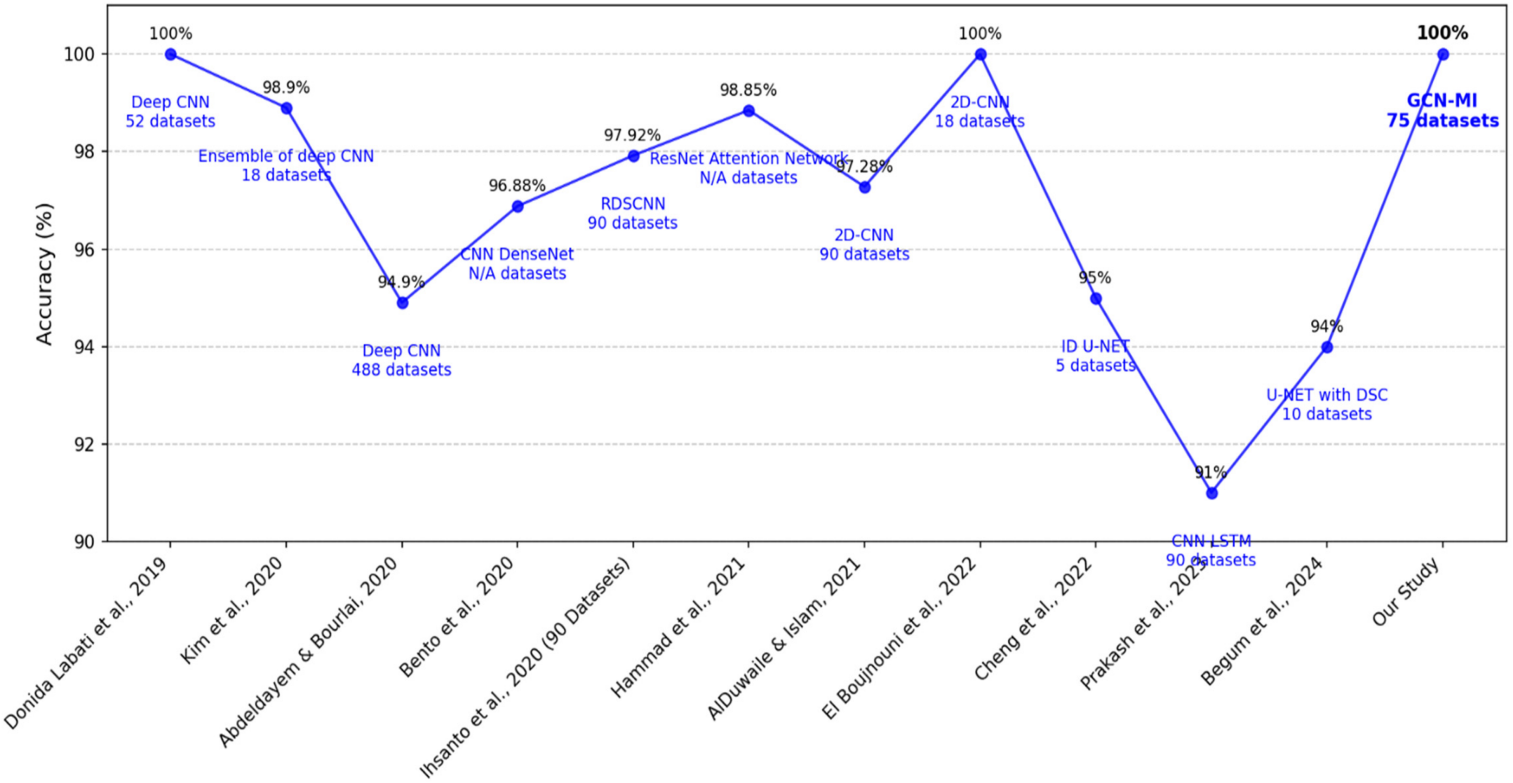
Comparison of performance of different DL methods used for human identification.

The GCN-MI model achieved 100% accuracy, outperforming all other methods in Table 10. Notably, methods such as the Deep CNN (45) gained 94.90%, while the Ensemble of Deep CNNs (46) reached 98.90%. Although some methods, such as 2D-CNN (47) and Deep CNN (48), also reported 100% accuracy, these were evaluated on smaller datasets, limiting their generalizability. Moreover, there are fundamental differences in methodology, computational efficiency, and attack resistance. Whereas El Boujnouni et al. and Donida Labati et al. employ a CNN-based architecture to extract features directly from ECG signals in a grid format, our approach introduces a novel graphical representation based on Graph Convolutional Networks (GCN) and Mutual Information (MI), allowing for a more precise modeling of interdependencies between the 12 ECG leads. This strategy optimizes feature selection and reduces computational overhead compared to deep CNN architectures, which require large amounts of data and high processing capacity, as illustrated in Figure 11. Our model provides significantly higher security. Since an attack would require replicating coherent electrical activity across 12 leads simultaneously, it is far more resistant to spoofing attempts (fabricating fake ECG signals) and impersonation attacks (fraudulently assuming another individual's identity) than a system based on a single ECG signal.

**Table 10:** List of recent studies on ECG-based human identification using DL techniques.

Reference	Method	Number of datasets	Accuracy (%)
Donida Labati et al. (48)	Deep CNN	52	100
Kim et al. (46)	Ensemble of deep CNN	18	98.90
Abdeldayem and Bourlai (45)	Deep CNN	488	94.90
Bento et al. (49)	CNN DenseNet	Not Specified	96.88
Ihsanto et al. (50)	RDSCNN	48	97.92
		90	98.89
AlDuwaile and Islam (51)	ResNet Attention Network	Not Specified	98.85
		Not Specified	99.27
AlDuwaile and Islam (52)	2D-CNN	90	97.28
El Boujnouni et al. (47)	2D-CNN	18	100
Cheng et al. (53)	ID U-NET	5	95
Prakash et al. (14)	CNN-LSTM	90	91
Begum et al. (54)	U-NET with DSC	10	94
**Our Study**	**GCN-MI**	**75 (12 leads and 2,570 samples)**	**100**

2 **Comprehensive Dataset Utilization**

The GCN-MI model was trained and validated on 75 datasets comprising 12 leads and 2,570 samples, providing a robust evaluation framework. This contrasts with many other studies that relied on smaller datasets. Cheng et al. (53) utilized only five datasets, while El Boujnouni et al. (47) used 18. The larger dataset used in this study demonstrates the scalability and reliability of the GCN-MI model in handling diverse data.

B. **Innovations Comparative**
  1 **Feature Selection Optimization:** The GCN MI framework's use of MI indices ensures a more effective feature representation, unlike methods such as CNN- LSTM (14), which primarily focuses on temporal patterns without explicitly modeling inter-lead dependencies.
  2 **Graph-Based Framework Advantages:** Unlike conventional deep learning methods, such as CNNs and U-Nets, the GCN-MI model integrates a graph-based learning framework, leveraging mutual information indices to model both linear and nonlinear dependencies between ECG leads. This innovative approach enhances the model's ability to capture complex relationships, enabling superior performance even with limited training data.
  3 **Scalability and Efficiency:** While methods like ResNet Attention Network (51) achieve high accuracy (99.27%), their performance is not tested on large-scale datasets or with graph-based dependencies.
  4 **Generalization:** Table 6. demonstrates accuracy differences of the GCN-MI model across k-fold value splits. Results suggest that model complexity and data availability significantly impacts performance. The results show that higher k-fold values, such as Fold-75 and Fold-70, with GCN-MI-10 achieve higher accuracy, leading to improved generalization —a challenge often encountered by traditional CNN-based methods. This trend suggests that deeper GCN architectures can learn more robust graph representations when more training data is available, leading to improved classification performance.

On the other hand, at lower k-fold values (Fold-60, Fold-65), simpler models such as GCN-MI-5 showed comparable or slightly better accuracy than deeper architectures. This indicates that increasing model complexity in scenarios with limited training data may lead to overfitting, reducing generalization performance. These results highlight the importance of selecting an appropriate model based on dataset size and application-specific constraints.

C. **Computational Efficiency:** While the proposed GCN-MI approach demonstrates superior accuracy in ECG-based biometric identification, a detailed computational comparison with existing methods remains limited. GCN-MI's computational efficiency stems from its ability to capture both linear and nonlinear dependencies between ECG leads using Mutual Information (MI), thereby optimizing feature selection and reducing redundancy.

The results in Table 1 demonstrate that unlike conventional methods such as Pearson correlation or Euclidean distance, which primarily detect linear relationships, our approach employs Mutual Information (MI), capable of capturing nonlinear dependencies between ECG leads. This results in a more robust and discriminative feature space, thereby improving classification accuracy and generalization.

Another significant factor contributing to the superior performance of our model is the use of 12-lead ECG signals, as opposed to single-lead ECG data, which was predominantly used in previous studies. By incorporating multiple leads, our approach maximizes the physiological information available, thereby reducing the risk of misclassification due to variations in a single lead and enhancing the model's ability to distinguish between subjects with more excellent reliability. This also has significant security implications, as single-lead ECG-based authentication systems are more susceptible to spoofing attacks, where an attacker can attempt to replicate the biometric signal using AI-generated or synthetic ECG waveforms. The multi-lead representation used in our approach makes such attacks significantly more difficult, as it requires generating a coherent and synchronized 12-lead ECG pattern, which is substantially more complex than forging a single-lead signal. Additionally, Table 1 shows that while previous studies have reported high accuracy values, they were often based on datasets such as PhysioNet, ECG-ID, or PTBDB, which contain a more limited diversity of subjects or lower inter-lead variability compared to INCART. Since no prior studies have utilized INCART for ECG-based authentication, a direct comparison with past models on the same dataset was not feasible. To ensure a meaningful comparison, we evaluated our results against previous studies that employed ECG authentication methods with a reduced number of leads, enabling us to assess the impact of using a multi-lead approach on both accuracy and security. As shown in Table 1, our model achieves 100% accuracy, surpassing previous works that ranged between 80% and 99.85%, while also demonstrating higher resilience against forgery attempts. The improvements introduced by our approach—namely, multi-lead ECG representation, GNN-based learning, and MI-driven feature extraction—offer a clear advantage over conventional single-lead methods. Furthermore, the graph-based structure of GCN-MI reduces the number of layers required for learning compared to CNN-based models, thereby improving scalability while maintaining high accuracy. These findings highlight the novelty and effectiveness of the GCN-MI model, reinforcing its practical applicability in secure biometric authentication while addressing the inherent vulnerabilities present in traditional ECG-based approaches. However, while GCN-MI is computationally more efficient than deep CNN architectures, it remains more complex than simple distance-based similarity measures. Table 11. presents a comparative analysis of feature extraction methods, accuracy, and estimated computational cost across different ECG-based authentication techniques.

**Table 11:** Computational comparison of ECG-based identification methods.

Method	Parameter	Accuracy (%)	Computational cost
Euclidean Distance (11)	Euclidean Distance	80.00%	Low
Pearson Correlation (15)	Pearson Correlation	98.30%	Moderate
Cosine Similarity (13)	Cosine Similarity	98.71%	Moderate
CNN-ResNet (51)	CNN with Attention	99.27%	High
**Proposed GCN-MI Approach**	**GCN-MI**	**100** **.** **00%**	**Moderate-High**

The main differences in computational efficiency between the GCN-MI model and earlier methods can be summarized as follows:

• **Consumed Training Time:** Compared to CNNs and RNNs, GCNs capture relationships between ECG leads with fewer layers, reducing overall training time but increasing per-iteration complexity**.**
• **Hardware Utilization:** Unlike CNNs, which benefit from GPU parallelization, GCNs require specialized graph-processing optimizations.
• **Scalability:** While CNNs struggle with capturing dependencies across long ECG sequences, GCNs provide better scalability by structuring data into a node-based format, improving adaptation to multi-lead ECG authentication.
• **Robustness:** MI techniques enhance the ability to learn invariant representations, making the approach less susceptible to noise and physiological variations, which are common limitations in traditional models.
• **Memory and Computational Load:** CNNs and RNNs require extensive GPU memory due to high-dimensional convolutions and recurrent processing. However, GCNs optimize computation by leveraging sparse adjacency matrices, making them more efficient for graph-based ECG analysis.

Future work should provide a more extensive computational performance analysis, including processing time, memory consumption, and scalability under real-world constraints.

D. **Usability and Practical Considerations:** The GCN-MI model's ability to achieve perfect accuracy on a large and diverse dataset positions it as a reliable solution for secure biometric authentication. Its graph-based learning and mutual information integration innovations provide a robust foundation for real-world applications, particularly in sectors where reliability and scalability are critical. However, its usability presents notable challenges in Table 12. compared to other biometric authentication methods in the literature. The reliance on a 12-lead ECG measurement system, though highly secure and resilient against spoofing, introduces practical limitations regarding user convenience and real-world applicability. Unlike fingerprint or facial recognition systems, which are widely adopted due to their ease of use and minimal setup requirements, multi-lead ECG authentication demands precise electrode placement and a controlled environment, making it less accessible for daily authentication scenarios such as smartphone unlocking or workstation logins. Compared to single-lead ECG authentication systems, commonly integrated into wearable devices such as smartwatches, a 12-lead system requires multiple electrode placements on the body, limiting its feasibility for continuous or on-the-go authentication. Although single-lead approaches sacrifice some accuracy in exchange for increased convenience, they offer a more practical solution for real-time and mobile applications. Nevertheless, the GCN-MI approach compensates for this limitation by significantly enhancing security and robustness, particularly in high-risk applications where forgery resistance and precision are paramount, such as healthcare authentication and secure access control in critical infrastructure and financial transactions.

**Table 12:** Usability comparison of ECG-based biometric methods.

Method	Number of leads required	Usability	Security level
Fingerprint Recognition	–	High	Moderate
Facial Recognition	–	High	Moderate
Single-Lead ECG (Wearable Devices)	1	Very High	High
**(Proposed GCN-MI)**	**12**	**Low-Moderate**	**Very High**

Our model has been designed to optimize computational efficiency, ensuring that the GCN framework can process multi-lead ECG data efficiently without incurring excessive resource consumption. While some applications, such as mobile banking, primarily rely on single-lead ECG devices (e.g., smartwatches or portable ECG monitors), our approach is particularly suited for high-security applications such as border control, forensic authentication and critical access control, where security is prioritized over convenience.

In healthcare settings, ECG-based biometric authentication can be used to secure patient identity verification, ensuring that electronic health records (EHRs) are accessed only by authorized individuals. This is particularly useful in hospital environments, where traditional passwords or biometric systems may not be feasible due to hygiene concerns. The results suggest that GCN-MI-10 and GCN-MI-15, which perform best with larger datasets, may be effective in hospital databases where a large number of patient ECG signals are available.

From a cybersecurity perspective, ECG-based authentication can enhance multi-factor authentication (MFA) frameworks, providing an additional layer of security against AI-driven attacks. As deepfake biometric spoofing techniques continue to advance, ECG biometrics could serve as a physiological authentication method that is harder to replicate compared to visual biometrics.

Although the proposed GCN-MI models demonstrate high classification accuracy, further improvements could enhance their robustness and adaptability across various biometric authentication scenarios.

One potential improvement is data augmentation. ECG signals can vary significantly due to environmental factors, electrode placement, and individual physiological differences. To improve model generalization, techniques such as random noise injection, time warping, and varying sampling rates can be used to simulate real-world variations in ECG signals, thereby reducing overfitting and enhancing model robustness.

Another optimization strategy is to explore additional physiological signals that complement ECG authentication. While the ECG alone provides a robust biometric signature, integrating photoplethysmography (PPG) or electromyography (EMG) signals could further enhance authentication reliability. Multi-modal biometric authentication could increase security while maintaining usability.

Additionally, improving the scalability of our model to enable real-time authentication for secure facility access or high-security transactions could be a valuable enhancement. Exploring edge computing approaches to process ECG signals locally, without relying on cloud-based processing, could reduce latency and enhance privacy in real-world deployments.

E. **Robustness of the Model to Cardiac Pathologies:** An essential consideration for ECG-based biometric authentication is the potential impact of cardiac pathologies, whether chronic (e.g., arrhythmias, ischemic heart disease) or transient (e.g., stress-induced variations, medication effects, electrolyte imbalances). Since our method relies on 12-lead ECG signals, significant changes in the heart's electrical activity—whether temporary or permanent—could alter the biometric signature and impact authentication accuracy. However, the multi-lead structure of our GCN-MI model inherently enhances its resilience compared to single-lead approaches, as it captures spatial correlations between leads. While minor variations in individual leads may not significantly impact performance, widespread pathological alterations affecting the entire cardiac cycle could present challenges, potentially requiring periodic recalibration of the system. At this stage, the model has been trained and validated using datasets composed of healthy subjects, and its robustness in the presence of cardiac diseases remains an open question that will be explored in future work.

To thoroughly assess the impact of cardiac conditions, future research will focus on validating the system using datasets that include individuals with diverse cardiac pathologies, ensuring a more comprehensive evaluation. However, this investigation relies on the availability of large-scale, high-quality 12-lead ECG databases that contain both healthy subjects and individuals diagnosed with various cardiovascular conditions. Strategies like adaptive learning techniques to adjust to gradual physiological changes, re-enrollment mechanisms for updating ECG templates, and anomaly detection models to differentiate between natural variability and authentication errors will be explored. While our results demonstrate that the proposed method is already highly effective in controlled conditions, extending validation to datasets with pathological cases will further reinforce its robustness and applicability in a broader range of real-world scenarios.

Given these advancements, our research proposes an innovative deep learning-based ECG authentication system that integrates 12-lead ECG readings with advanced graph convolutional network architectures to enhance robustness and scalability. We aim to improve authentication accuracy and generalizability in real-world applications by leveraging state-of-the-art deep learning methodologies and multi-lead ECG signals.

Our study concludes that in clinical or biometric authentication systems where data availability is constrained, GCN-MI-5 could be the preferred choice due to its robustness with smaller datasets. For high-security systems with abundant data, GCN-MI-10 or GCN-MI-15 could offer improved accuracy and reliability by capturing more complex patterns in ECG signals. Lightweight models, such as GCN-MI-5, are more suitable for deployment on wearable devices or mobile systems with limited computational resources. On the other hand, GCN-MI-10 and GCN-MI-15 can be utilized in server-based systems for applications that require higher computational capabilities.

The proposed optimizations, including data augmentation, multimodal biometric fusion, and edge computing integration, represent promising directions for future research. Future studies could investigate how to optimize the balance between computational efficiency and biometric robustness, ensuring that 12-lead ECG authentication remains secure while being adaptable to various real-world constraints. Additionally, further research is needed to evaluate the performance of GCN-MI models under adversarial conditions, ensuring their robustness against potential spoofing attacks.

## 6 Conclusions

This study introduces a novel Graph Convolutional Network (GCN) framework, combined with Mutual Information (MI), to enhance ECG-based biometric authentication. The proposed GCN-MI approach leverages 12-lead ECG signals, providing a richer and more robust representation of biometric data than traditional single-lead methods.

Using a graph-based modeling approach, our method effectively captures the spatial and statistical dependencies between ECG leads, thereby enhancing classification accuracy and system robustness. The integration of MI into the GCN architecture enables a more informative and adaptive feature extraction process, surpassing conventional methods that rely on predefined distance metrics.

Our experimental results demonstrate that the GCN-MI model achieves 100% accuracy on the INCART dataset, outperforming previous deep learning-based ECG authentication methods. Unlike other models that process ECG signals as independent time series, our approach models them as graph-structured data, enabling the system to learn intricate inter-lead relationships.

The use of a multi-lead approach also enhances security by making the system more resistant to spoofing attacks, as replicating a full 12-lead ECG pattern is significantly more challenging than replicating a single-lead signal.

**In summary, the main contributions of this research include the following:**

• Innovation in the Design Approach: We introduce a novel GCN-MI framework that models ECG signals as graph-structured data and exploits mutual information to capture both linear and nonlinear interdependencies among 12-lead ECG signals. This enhances the system's ability to extract distinctive biometric patterns.
• Performance Improvements and Theoretical Advancements: Our approach achieves state-of-the-art performance in ECG-based authentication, demonstrating superior accuracy compared to traditional methods. The use of MI indices enhances feature representation by quantifying complex dependencies, thereby offering a more effective biometric authentication model.
• Computational Efficiency and Scalability: The proposed MI-based edge construction mechanism in the GCN framework reduces computational complexity while maintaining high performance. Our method is designed to scale efficiently for real-world applications, making it feasible for large-scale biometric authentication systems.
• Enhanced Security and Practical Applicability: Unlike single-lead ECG authentication models, our multi-lead approach offers greater resistance to impersonation attacks. This has significant implications for secure authentication in banking, healthcare, and personal security applications, where identity verification must be both accurate and resilient.

By demonstrating the advantages of the GCN-MI approach, this study lays the foundation for future research in graph-based biometric authentication, offering a more reliable and secure alternative to traditional ECG-based methods. These findings pave the way for the development of advanced biometric security systems, addressing key challenges in authentication, generalization, and robustness against adversarial attacks.

## Data Availability

The original contributions presented in the study are included in the article/Supplementary Material, further inquiries can be directed to the corresponding authors.
